# Causal Analysis Between Gut Microbes, Aging Indicator, and Age‐Related Disease, Involving the Discovery and Validation of Biomarkers

**DOI:** 10.1111/acel.70057

**Published:** 2025-04-09

**Authors:** Chunrong Lu, Xiaojun Wang, Xiaochun Chen, Tao Qin, Pengpeng Ye, Jianqun Liu, Shuai Wang, Weifei Luo

**Affiliations:** ^1^ AIage Life Science Corporation Ltd., Guangxi Free Trade Zone Aisheng Biotechnology Corporation Ltd. Nanning Guangxi China; ^2^ Guangxi Key Laboratory of Longevity Science and Technology Nanning Guangxi P.R. China; ^3^ State Key Laboratory for Animal Disease Control and Prevention, College of Veterinary Medicine, Lanzhou Veterinary Research Institute Chinese Academy of Agricultural Sciences, Lanzhou University Lanzhou Gansu China

**Keywords:** aging, gut microbiota, machine learning, Mendelian randomization

## Abstract

The influence of gut microbes on aging has been reported in several studies, but the mediating pathways of gut microbiota, whether there is a causal relationship between the two, and biomarker screening and validation have not been fully discussed. In this study, Mendelian Randomization (MR) and Linkage Disequilibrium Score Regression (LDSC) are used to systematically investigate the associations between gut microbiota, three aging indicators, and 14 age‐related diseases. Additionally, this study integrates machine learning algorithms to explore the potential of MR and LDSC methods for biomarker screening. Gut microbiota is found to be a potential risk factor for 14 age‐related diseases. The causal effects of gut microbiota on chronic kidney disease, cirrhosis, and heart failure are partially mediated by aging indicators. Additionally, gut microbiota identified through MR and LDSC methods exhibit biomarker properties for disease prediction (average AUC = 0.731). These methods can serve as auxiliary tools for conventional biomarker screening, effectively enhancing the performance of disease models (average AUC increased from 0.808 to 0.832). This study provides evidence that supports the association between the gut microbiota and aging and highlights the potential of genetic correlation and causal relationship analysis in biomarker discovery. These findings may help to develop new approaches for healthy aging detection and intervention.

## Graphical abstract

A comprehensive causal effect analysis was used to illustrate the associations between gut microbiota, aging indicators, and age‐related diseases. This study provides evidence that supports the association between the gut microbiota and aging and highlights the potential of genetic correlation and causal relationship analysis in biomarker discovery.

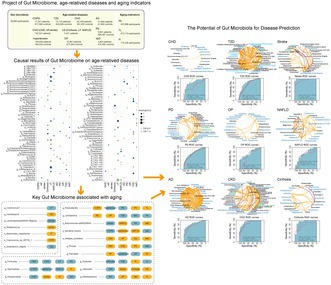

## 1 Introduction

Aging is typically accompanied by cellular aging, immune senescence, organ dysfunction, and age‐related diseases, and is associated with systemic chronic inflammation. Chronic inflammation accelerates immune cell aging, leading to impaired clearance of senescent cells and inflammatory factors, resulting in a vicious cycle of inflammation and aging. The maintenance of high levels of inflammatory factors in various organs can lead to organ damage and age‐related diseases (Li, Li, et al. 2023). Consequently, numerous diseases are associated with aging and inflammation, including cardiovascular disease (CVD), type 2 diabetes (T2D), Alzheimer's disease (AD), Parkinson's disease (PD), chronic kidney disease (CKD), chronic obstructive pulmonary disease (COPD), and liver disease (Rossiello et al. 2022; Marta and Costantino 2020; Collier et al. 2017). For example, aging and inflammation are considered key triggers for CVD (Liberale et al. 2022). As research has progressed, the human gut microbiome has been found to play a critical role in human health. Gut microbiota dysbiosis is thought to be associated with the development and progression of various diseases (Wu et al. 2021) and has been listed as a hallmark of aging (Carlos et al. 2023). However, most studies on the relationship between the gut microbiota and age‐related diseases have been limited to observational studies (Chen et al. 2021; Lecamwasam et al. 2020; Marchal 2024). The causal relationship between the gut microbiota and age‐related diseases has not been fully elucidated.

Aging indicators, such as telomere length (TL), the frailty index (FI) and facial aging (FA), are considered to be essential components of aging characteristics (Rossiello et al. 2022; Franco et al. 2022) and can be used to assess accelerated aging (Jylhävä et al. 2017; Chen et al. 2023). Chen et al. (2023) analyzed genetic variations associated with aging indicators and overweight, confirming a causal relationship between overweight and accelerated aging and reduced life expectancy. In other studies on aging indicators and age‐related diseases (Chen and Zhan 2021; Gurung et al. 2021), TL was found to have genetic associations with age‐related diseases, and an elevated FI was bidirectionally associated with increased CVD risk (Xu, Jia, et al. 2024). Furthermore, some studies have attempted to explore the relationships between aging indicators and gut microbiota from a genetic and phenotypic perspective. Specifically, these findings reveal a bidirectional causal relationship between gut microbiota and FI (Bo et al. 2024), as well as a significant causal relationship with FA (Chen, Che, et al. 2024), and providing some evidence for the relationships among aging indicators, gut microbiota, and age‐related diseases. However, it is unclear whether these indicators mediate the relationship between gut microbiota and age‐related diseases.

In recent years, statistical methods based on genome‐wide association studies (GWAS) have been employed to estimate correlations and causal relationships between traits, especially to analyze the interplay between gut microbiota and other traits, including diseases and metabolites (Wang et al. 2023; Yan et al. 2024). Linkage disequilibrium score regression (LDSC) can be used to assess genetic correlations between traits from GWAS summary statistics without bias due to sample overlap. Furthermore, Mendelian randomization (MR) utilizes genetic variants as instrumental variables to explore causal associations between exposures and outcomes, offering advantages in mitigating measurement errors, controlling for confounding factors, and reducing reverse causation bias. Consequently, the application of LDSC and MR enables the elucidation of deeper relationships between traits from the perspectives of genetic correlation and causality.

Given the ambiguous connection between specific gut bacteria taxa and human aging, we used LDSC to estimate the genetic correlation between the gut microbiota and aging indicators on multiple age‐related diseases. We also conducted five MR methods to explore causal relationships between the gut microbiota and aging indicators on multiple age‐related diseases. In addition, the correlated microbes identified by these two methods were used as novel microbial markers for the corresponding diseases to construct machine learning models, and the efficacy of these markers in disease prediction was evaluated. The findings of this study may provide valuable insights for future research on the genetic underpinnings and biological interventions in aging related to the complex features associated with the gut microbiota and aging.

## 2 Methods

### 2.1 Study Design

**FIGURE 1:**
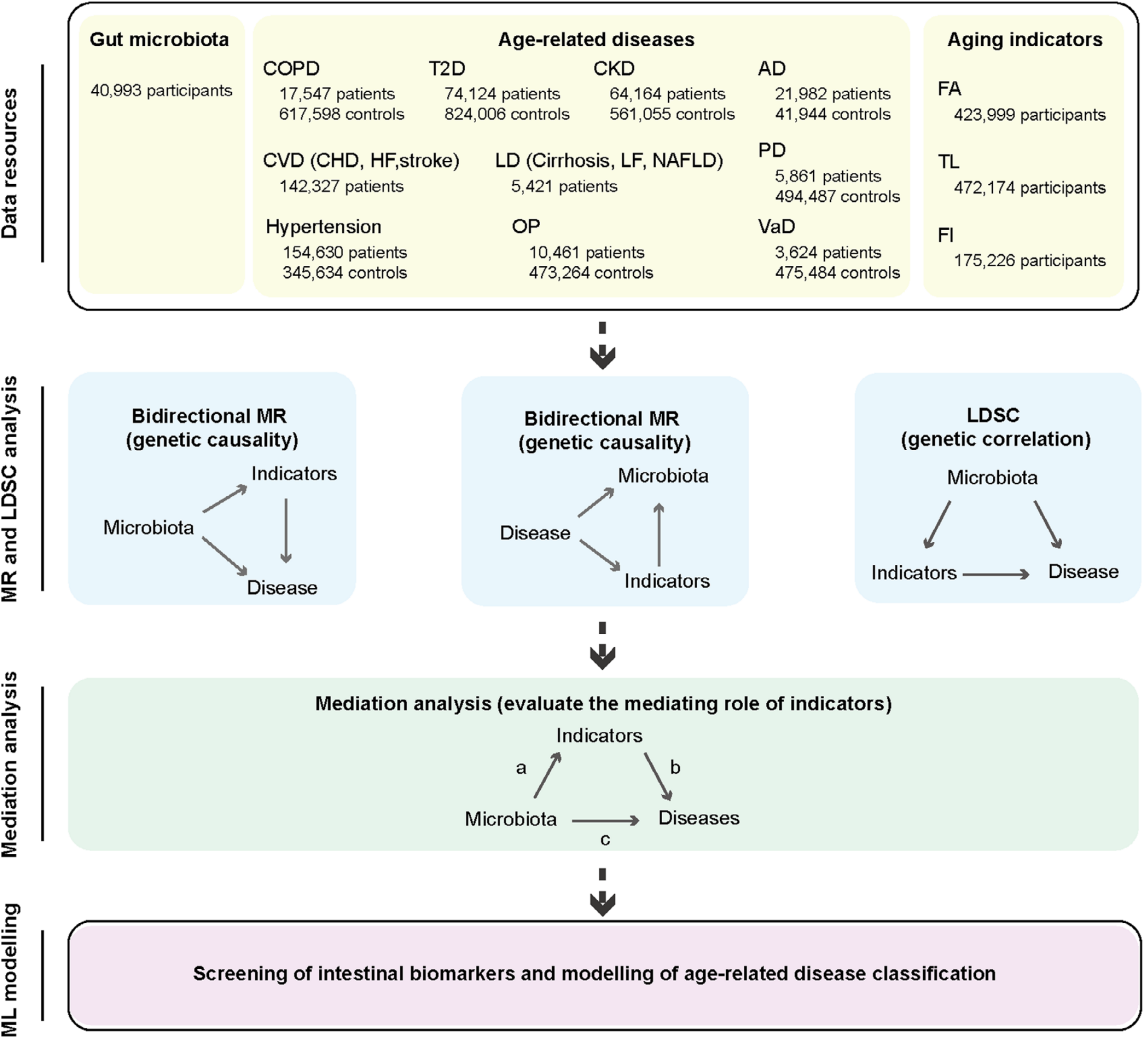
Flowchart illustrating the bidirectional MR study design for investigating the causal associations between gut microbiota, aging indicators, and age‐related diseases. AD, Alzheimer's disease; CHD, coronary atherosclerotic heart disease; CKD, chronic kidney disease; COPD, chronic obstructive pulmonary disease; FA, facial aging; FI, frailty index; HF, heart failure; LDSC, Linkage Disequilibrium Score Regression; LF, liver fibrosis; ML, machine learning; MR, Mendelian Randomization; NAFLD, nonalcoholic fatty liver disease; OP, osteoporosis; PD, Parkinson's disease; T2D, type 2 diabetes; TL, telomere length; VaD, vascular dementia.

A comprehensive causal effect analysis was used to illustrate the associations between gut microbiota, aging indicators, and age‐related diseases. These analyses are based on the two‐sample MR method, with gut microbiota set as exposures and 14 age‐related diseases and three aging indicators as outcomes. The fourteen age‐related diseases include T2D, CKD, Hypertension, Alzheimer's disease (AD), Parkinson's disease (PD), Osteoporosis (OP), Vascular dementia (VaD), CVD, including coronary heart disease (CHD), heart failure (HF), and stroke, COPD, and liver disease (LD), including cirrhosis, liver fibrosis (LF), and nonalcoholic fatty liver disease (NAFLD). The three aging indicators include FA, FI, and TL. Simultaneously, reverse MR analyses were conducted to explore the effects of age‐related diseases and aging indicators on gut microbiota (Figure 1). Besides large‐scale MR analysis, we also conducted a LDSC to probe genetic correlations between age‐related diseases, aging indicators, and gut microbiota, respectively. In addition, to discover whether aging indicators serve as mediators between gut microbiota and age‐related diseases, mediation analyses were employed. Finally, to investigate the biomarker characteristics of the gut microbiome identified through genetic correlation and causal relationship analyses, we employed machine learning algorithms to construct corresponding disease prediction models and evaluated their predictive performance.

### 2.2 Exposure and Outcome Data Source

Summary statistics of gut microbiota are derived from four datasets (Liu et al. 2024; Chen, Chen, et al. 2024; Chen, Zhang, et al. 2024): multiple cohorts from MiBioGen consortium (Kurilshikov et al. 2021), Dutch Microbiome Project (DMP) (Lopera‐Maya et al. 2022), German individuals (Rühlemann et al. 2021), FINRISK 2002 (FR02) cohort (Qin et al. 2022). The MiBioGen consortium cohort comprises 21 subordinate cohorts and has 18,340 participants from various regions, such as Europe, Asia, Africa, and America. This gut microbiota cohort includes 211 taxa (131 genera, 35 families, 20 orders, 16 classes, and 9 phyla), but 15 unknown taxa were incorporated into it. The Dutch Microbiome Project includes 7,738 participants from the Netherlands; 207 taxa and 205 functional pathways were identified by shotgun metagenomic sequence in stool samples. We only used bacteria from the phylum to species level that are not included in the MiBioGen cohort. The German individual cohort of Ruhlemann et al., consisting of 8956 participants, was identified by 16S rRNA sequence. The cohort identified 430 taxa from phylum to genus, in both abundance and prevalence forms. We only used bacteria in the abundance format that are not included in the MiBioGen cohort. The FR02 project, with 5959 Finnish participants, identified 473 taxa from phylum to species through fecal shotgun sequences. We only used bacteria that were not included in the MiBioGen cohort and had no unknown taxa. Summary statistics data of FA, FI, and TL are all of European ancestry and can be downloaded from the IEU Open GWAS database (Atkins et al. 2021).

The GWAS summary data of T2D were obtained from the Diagram Consortium, and all samples are from European ancestry (Mahajan et al. 2018). CKD summary data were derived from Matthias Wuttke's research (Wuttke et al. 2019), and all samples are from European ancestry. COPD summary data were come from Saori Sakaue's research (Sakaue et al. 2021), a cohort of participants from Europe and East Asia. CVD includes three types: CHD, HF, and stroke. Among these three types, CHD data were sourced from Majid Nikpay's study (Nikpay et al. 2015). HF data were sourced from Sonia Shah's study (Shah et al. 2020). Stroke data were sourced from Rainer Malik's study (Malik et al. 2018). All CVD datasets were from European descent. Summary statistics for Hypertension, AD (Zeng et al. 2023), PD, OP (Zhang et al. 2024), VaD, and LD were derived from the FinnGen dataset, all of which were of European descent. LD incorporates three diseases: cirrhosis, LF, and NAFLD. The AD summary data were derived from a study by Brian W Kunkle et al. (2019), which involved participants from Europe. Detailed characteristics of the data sources used in this study can be found in Table S1.

### 2.3 Instrument Variables Selection

First, the use of Instrument variables (IVs) in MR analysis shall satisfy three principles: (1) IVs have a strong correlation with exposures, (2) IVs are not correlated with confounders, (3) the exposure only affects the outcome through the IVs, without any other path. Second, single nucleotide polymorphisms (SNPs) show a significant correlation with gut microbiota under the condition of *p* < 1 × 10^−5^. SNPs have few associations with each other through linkage disequilibrium clumping (*r* ^2^ = 0.01; distance = 10.00 kb). SNPs show a strong association with gut microbiota and are free from weak instrument bias, with an *F*‐statistic > 10. For the aging indicators, we selected the SNPs with a *p*‐value of 5 × 10^−8^ as the threshold.

### 2.4 MR and Reverse MR Analysis

The MR and reverse MR analyses were performed using the same methods (In addition to the reverse MR Analysis between gut microbiota and hypertension, the threshold for hypertension was set at *p* < 5 × 10^−8^ because too many SNPS were retained after *p* < 1 × 10^−5^). After IVs were selected with the threshold depicted above, eligible ones were used to conduct MR analysis to estimate the causal effect between gut microbiota and 6 age‐related diseases and 3 aging indicators. We use five methods to perform MR analysis, including inverse variance weighted (IVW), weighted median, weighted mode, simple mode, and MR‐Egger. IVW was the principal method used to estimate the causal effect in this study; it included fixed‐effect IVW and random‐effect IVW. When heterogeneity existed, random‐effect IVW would be used. Otherwise, fixed‐effect IVW would be used.

### 2.5 LDSC Analysis

To strengthen the evidence of causal effect in MR analysis, LDSC was used to evaluate the heritability from GWAS summary statistics of age‐related diseases, aging indicators, and gut microbiota. Heritability estimates from LDSC can reveal the genetic correlation, and they are not affected by overlapping samples (Bulik‐Sullivan et al. 2015). Only the significant exposures and outcomes of MR were selected to conduct the LDSC analysis.

### 2.6 Sensitivity Analyses

Sensitivity analysis was performed to evaluate the reliability of the MR analysis, including pleiotropy and heterogeneity. In this study, Cochran's *Q* statistic was used to estimate heterogeneity, and the MR‐Egger regression intercept test was used to monitor the horizontal pleiotropy effect (*P*_intercept). In addition, the MR‐PRESSO method was used to detect SNP outliers with pleiotropic effects.

### 2.7 Mediation Analysis

After identifying the gut microbiota that show a causal effect on age‐related diseases, we sincerely wanted to know if this causal effect is affected by aging proxies. Thus, mediation analysis was subsequently applied. Causal effects are estimated by MR analysis. We defined the causal effect of gut microbiota on aging indicators as β1, the causal effect of aging indicators on age‐related diseases as β2, and the indirect effect (mediation effect) was calculated by the formula β1 × β2. Furthermore, we used the delta method to calculate standard errors (Carter et al. 2021).

### 2.8 Construction of Disease Prediction Machine Learning Models

LDSC and MR analyses identified gut microbial taxa exhibiting genetic correlations and causal relationships with various diseases. Given their strong association with disease risk, these taxa may serve as gut microbial markers for constructing disease prediction models. To further explore this possibility, we downloaded 16S rRNA sequencing data of gut microbiota from publicly available databases for the relevant diseases to serve as the dataset for model construction. Due to limitations in data completeness and availability, we obtained 16S sequencing data for nine out of the 14 diseases mentioned above. We then constructed machine learning models using the gut microbes identified by MR and LDSC as exhibiting significant causal relationships (at the genus level) as microbial markers (MR&LDSC method). In addition, we used fold change (FC) and *p*‐value to measure differential bacteria between disease and healthy groups. These differential bacteria were also used to construct disease classification models (FC method). Detailed information regarding the data and model construction process is provided in Appendix S1 (Supporting Information Material 1).

## 3 Results

### 3.1 Analysis of LDSC

We employed LDSC analysis to assess the genetic correlation among gut bacterial taxa, three aging indicators, and 14 age‐related diseases. In the analysis of gut microbiota on aging indicators and age‐related diseases, we obtained 3689 genetic correlation results (Figure S1, Table S2). Moreover, there were 42 genetic correlations between aging indicators and age‐related diseases (Figure S2, Table S2). LDSC showed 233 suggestive correlations among gut microbiota, aging indicators, and age‐related diseases (*p* < 0.05), as shown in Figures S1 and S2, Table S2.

### 3.2 Overview of Instrumental Variables for Age‐Related Diseases

Following a comprehensive series of quality control measures, the MiBioGen consortium identified 125, 230, 285, 501, and 1724 SNPs associated with gut microbiota at the phylum, class, order, family, and genus levels, respectively, at a significance level of *p* < 1 × 10^−5^ (Table S3). The results of other datasets are shown in Table S3. Furthermore, we identified 92, 17, and 233 SNPs associated with FA, FI, and TL, respectively, at a significance level of *p* < 5 × 10^−8^ (Table S4). For the MiBioGen consortium and aging indicators, a total of 2865 SNPs and 342 SNPs were chosen as instrumental variables (IVs), respectively.

### 3.3 Causal Effects of Gut Microbiota and Aging Indicators on Multiple Age‐Related Diseases

Utilizing the IVW method, we uncovered substantial evidence of a significant association among gut microbiota, aging indicators, and the risk of age‐related diseases.

#### 3.3.1 CDK

**FIGURE 2:**
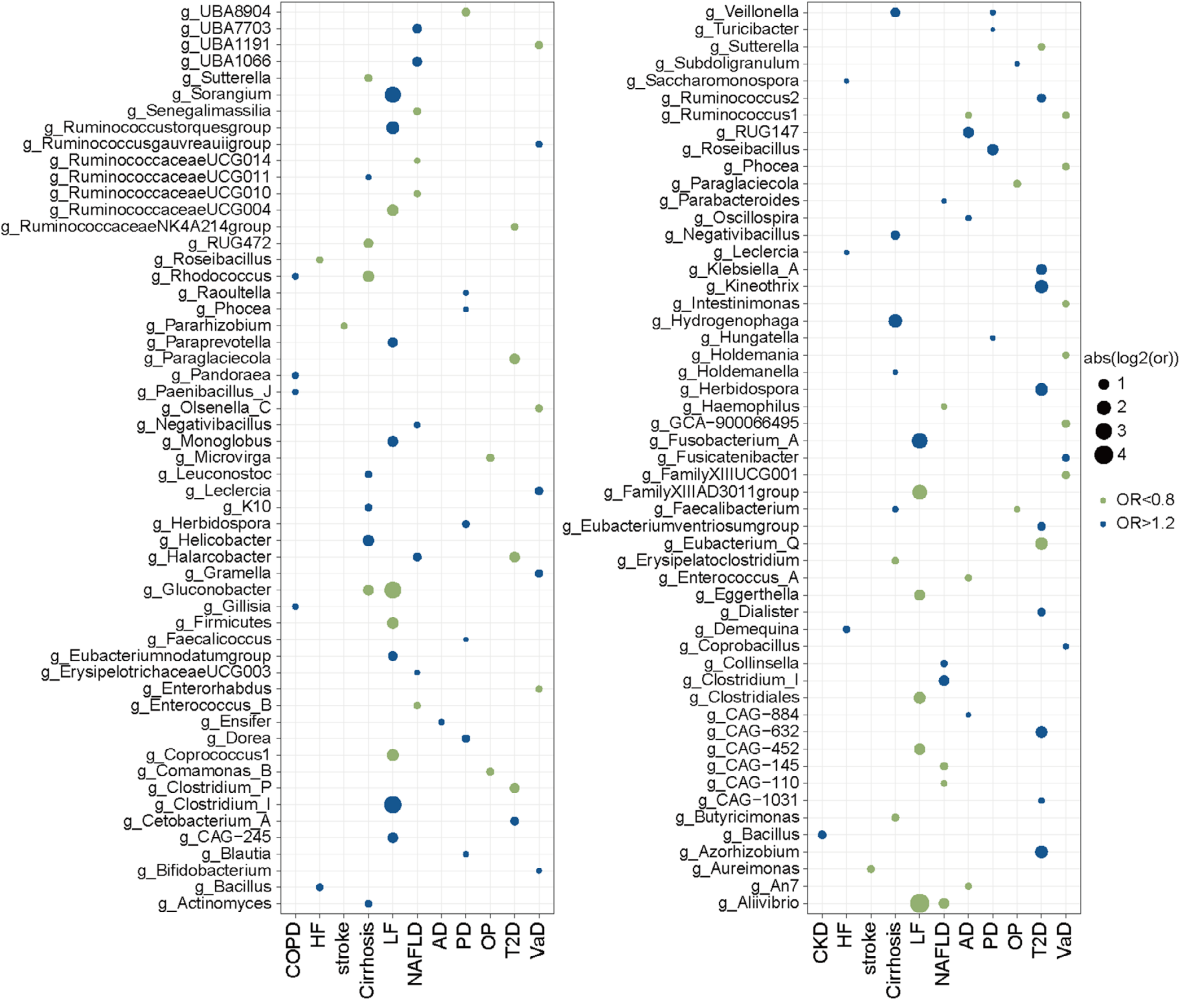
Mendelian randomization results of causal effects of gut microbiota on aging indicators and age‐related diseases. Associations with a *p*‐value < 0.05 are represented by dots. The size of the dots corresponds the effect size. Green dots signify a positive causal effect on the outcome, while blue dots indicate a negative causal effect. AD, Alzheimer's disease; CHD, coronary atherosclerotic heart disease; CKD, chronic kidney disease; COPD, chronic obstructive pulmonary disease; FA, facial aging; FI, frailty index; HF, heart failure; LF, liver fibrosis; NAFLD, nonalcoholic fatty liver disease; OP, osteoporosis; PD, Parkinson's disease; T2D, type 2 diabetes; TL, telomere length; VaD, vascular dementia.

We found that 60 gut microbiota taxa are associated with CDK, including 3 phyla, 4 classes, 1 order, 8 families, 18 genera, and 16 species (Figure 2, Figure S3, Table S5). For example, g_*Bacillus*, g_*Escherichia*, g_*Klebsiella*, g_*Leclercia*, g_*Phocea*, and g_*Ruminococcaceae UCG002* were associated with an increased risk of CDK, and g_*Firmicutes*, g_*Subdoligranulum*, g_*Family XIII ad3011 group*, g_*Veillonella*, g_*Lachnospiraceae UCG010*, and g_*Ruminococcaceae NK4A214 group* were associated with a decreased risk of CDK. Furthermore, TL (odds ratio, (OR)  =   0.875, 95% confidence interval (CI) = 0.817~0.938, *p* = 0.00016) and FA (OR = 0.688, 95% CI = 0.533~0.887, *p* = 0.00399) significantly decreased the incidence of CDK (Figure S4, Table S6).

#### 3.3.2 COPD

In evaluating the causal effect of microbiota on COPD, we found that a total of 59 gut microbiota (comprising 2 phyla, 1 class, 4 orders, 9 families, 21 genera and 22 species) were associated with COPD (Figure 2, Figure S3, Table S5). Specifically, s_*Faecalibacterium prausnitzii*_*E*, g*_Adlercreutzia*, and g*_Pandoraea* were linked to an increased risk of COPD, whereas s*_Bifidobacterium angulatum*, g*_Allisonella*, g*_Ensifer*, g*_Haemophilus*, g*_Proteus*, *and* g*_Victivallis* were associated with a decreased risk of COPD. Moreover, we did not find a significant association between FA, FI, or TL and the incidence of COPD (*p* > 0.05) (Figure S4, Table S6).g

#### 3.3.3 T2D

A total of 36 gut microbiota (including 5 phylum, 2 classes, 1 order, 4 families, and 19 genera and 9 species) were found to be associated with T2D (Figure 2, Figure S3, Table S5). The g_*Azorhizobium*, g_*Cetobacterium*_*A*, g*_Herbidospora*, and g_*Ruminococcus 2* increased the risk of T2D, while s_*Eubacterium*_*eligens*, s_*Bacteroides*_*caccae*, g*_Sutterella*, g*_Lactobacillus*, and g_*Ruminococcaceae NK4A214 group* were linked to a decreased risk of T2D. Moreover, we did not find a significant association between FA, FI, or TL and the incidence of T2D (*p* > 0.05).

#### 3.3.4 CVD

##### 3.3.4.1 CHD

In the MR analysis assessing the relationship between gut bacteria and CHD, we identified 17 gut microbiota taxa that were associated with an increased risk of CHD, including s_*Eubacterium rectale*, s_*Phocea massiliensis*, and g_*Phocea* (Figure 2, Figure S3, Table S5). Conversely, 18 gut microbiota taxa were associated with a decreased risk of CHD, including s_*Ruminococcus lactaris*, s_*Gordonibacter pamelaeae*, g_*Paraprevotella*, g_*Butyricicoccus*, and g_*Alloprevotella* (Figure 2, Figure S3, Table S5). Furthermore, among the three aging indicators examined, only the FI was significantly associated with an increased risk of CHD (OR = 1.622, 95% CI = 1.199–2.193, *p* = 0.0017; Figure S4, Table S6).

##### 3.3.4.2 HF

We found that 47 gut microbiota were associated with HF, including 2 classes, 5 orders, 7 families, and 20 genera and 13 species (Figure 2, Figure S3, Table S5). Specifically, s*_Ruminococcus_callidu,* s*_Raoultibacter_massiliensis*, g*_Leuconostoc*, g*_Escherichia*, and g*_Anaerostipes* were associated with a decreased risk of HF. Conversely, s_*Prevotella*_*copri*, s_*Eubacterium*_*eligens*, s*_Bacteroides_dorei*, s_*Alistipes*_*putredinis*, g*_Leclercia*, g*_Bacillus*, and g_*Flavonifractor* were linked to an increased incidence of HF. In the analysis of aging indicators, FI (OR = 1.423, 95% CI = 1.163~1.741, *p* = 0.0006) was significantly associated with an increased risk of HF (Figure S4, Table S6).

##### 3.3.4.3 Stroke

A total of 40 gut microbiota taxa (including 1 phylum, 4 classes, 2 orders, 3 families, 11 genera and 19 species) were found to be associated with Stroke (Figure 2, Figure S3, Table S5). Specifically, s_*Coprobacter*_*fastidiosus*, s_*Clostridium saudiense*, s_*Bacteroides*_*salyersiae*, g_*LachnospiraceaeNK4A136group*, and g_*Aureimonas* were associated with a decreased risk of stroke. Conversely, s_*Terrisporobacter othiniensis*, s_*Provencibacterium massiliense*, s_*Coprococcus*_*comes*, and g_*Streptococcus* were associated with an increased risk of stroke. Furthermore, none of the aging indicators (FA, FI, and TL) were significantly associated with the incidence of stroke (*p* > 0.05).

#### 3.3.5 LD

##### 3.3.5.1 Cirrhosis

Based on the results of the MR analysis, we found that several gut microbiota taxa were associated with the risk of cirrhosis. Specifically, s_*Bacteroides clarus*, s_*Eubacterium siraeum*, s_*Lactobacillus delbrueckii*, g_*Veillonella*, and g_*Veillonellae* were associated with an increased risk of cirrhosis (Figure 2, Figure S3, Table S5). Conversely, s_*Bifidobacterium adolescentis*, g_*Rhodococcus*, g_*Sutterella*, and s_*Parabacteroides merdae* were associated with a decreased risk of cirrhosis (Figure 2, Figure S3, Table S5). In addition, TL (OR = 0.568, 95% CI = 0.445~0.724, *p* = 4.96E‐06) decreased the risk of Cirrhosis (Figure S4, Table S6).

##### 3.3.5.2 LF

A total of 43 gut microbiota were causally associated with LF, including 1 phylum, 1 class, 2 orders, 4 families, 17 genera, and 18 species. In detail, s_*Dorea phocaeense*, s_*Clostridium tertium*, s_Bifidobacterium bifidum, g_*Firmicutes*, and g_*Family XIII AD3011group* were associated with a decreased risk of LF, while s_Ruminococcus bromii, s_*Monoglobus pectinilyticus*, and s_*Escherichia flexneri* were associated with an increased risk of LF (Figure 2, Figure S3, Table S5). No aging indicators were associated with the incidence of LF (*p* > 0.05).

##### 3.3.5.3 NAFLD

Twenty gut microbiota were associated with an increased risk of NAFLD, including s_*Kandleria vitulina*, s_*Klebsiella pneumoniae*, s_*Oxalobacter*_*formigenes*, s_*Phocea massiliensis*, s_*Ruminococcus*_*bromii*, g_*Parabacteroides*, g_*Oxalobacter*, and g_*Clostridium*_*I*, while s_*Ruminococcus*_*lactaris*, s_*Bacteroides*_*intestinalis*, g_*Aliivibrio*, and g_*Haemophilus* were associated with a decreased risk of NAFLD (Figure 2, Figure S3, Table S5). Additionally, no aging indicators were associated with the incidence of LF (*p* > 0.05) (Figure S4, Table S6).

#### 3.3.6 Hypertension

A total of 53 gut microbiota taxa (including 2 phyla, 3 classes, 2 orders, 7 families, 27 genera and 12 species) were found to be associated with Hypertension (Figure 2, Figure S3, Table S5). Specifically, g_*LachnospiraceaeNC2004group*, g_*LachnospiraceaeNK4A136group*, g_*RuminococcaceaeUCG010*, and g_*Ruminiclostridium6* were associated with a decreased risk of Hypertension. Conversely, s_*Kandleria vitulina*, s_*Haemophilus*_*parainfluenzae*, s_*Butyrivibrio*_*crossotus*, g_*Oxalobacter*, and s_*Bacillus velezensis* were associated with an increased risk of Hypertension. Furthermore, none of the aging indicators (FA, FI, and TL) were significantly associated with the incidence of Hypertension (*p* > 0.05).

#### 3.3.7 Ad

We found that 50 gut microbiota were associated with AD, including 5 phyla, 4 classes, 3 orders, 6 families, and 13 genera and 19 species (Figure 2, Figure S3, Table S5). Specifically, s*_Pseudomonas aeruginosa*, s_*Lachnospira rogosae*, s_*Coprobacillus cateniformis*, and *s_Bacteroides faecis* were associated with a decreased risk of AD. Conversely, s*_Ruminococcus callidus*, s_Parabacteroides distasonis, s_*Bifidobacterium adolescentis*, s_*Bifidobacterium infantis*, and *s_Alistipes putredinis* were linked to an increased incidence of AD. Additionally, no aging indicators were associated with the incidence of AD (*p* > 0.05) (Figure S4, Table S6).

#### 3.3.8 PD

In evaluating the causal effect of microbiota on PD, we found that a total of 51 gut microbiota (comprising 4 phyla, 5 classes, 3 orders, 3 families, 23 genera and 12 species) were associated with PD (Figure 2, Figure S3, Table S5). Specifically, s_*Pseudomonas aeruginosa*, s_*Faecalicoccus pleomorphus*, s_*Bifidobacterium*_*bifidum*, s_*Bacteroides*_*plebeius*, g_*Lactobacillus*, g_*Faecalicoccus*, and g*_Phocea* were linked to an increased risk of PD, whereas s*_Dorea_longicatena,* s*_Bifidobacterium ruminantium,* g*_Enterorhabdus, and* g_*Escherichia* were associated with a decreased risk of PD. In addition, TL (OR = 1.155, 95% CI = 1.012~1.318, *p* = 0.033) increased the risk of PD (Figure S4, Table S6).

#### 3.3.9 OP

A total of 54 gut microbiota taxa (including 3 phyla, 4 classes, 4 orders, 8 families, 19 genera and 16 species) were found to be associated with OP (Figure 2, Figure S3, Table S5). Specifically, s_*Terrisporobacter othiniensis*, s_*Staphylococcus aureus*, g_*Roseburia*, and g_*Microvirga* were associated with a decreased risk of OP. Conversely, s_*Prevotella buccae*, s_*Absiella dolichum*, g_*Subdoligranulum*, and g_*Prevotella* were associated with an increased risk of OP. Furthermore, none of the aging indicators (FA, FI, and TL) were significantly associated with the incidence of OP (*p* > 0.05).

#### 3.3.10 VaD

In evaluating the causal effect of microbiota on VaD, we found that a total of 46 gut microbiota (comprising 1 phylum, 1 class, 5 orders, 5 families, 19 genera and 15species) were associated with VaD (Figure 2, Figure S3, Table S5). Specifically, s_*Bifidobacterium pseudocatenulatum,* s*_Alistipes_putredinis,* g*_Leclercia,* g*_Clostridium*, and s*_Bifidobacterium catenulatum* were linked to an increased risk of VaD, whereas s_*Phocea massiliensis*, s_*Enorma massiliensis*, g_*Ruminococcus1*, g_*Lachnospiraceae*, g_*Prevotella7*, and s_*Kandleria vitulina* were associated with a decreased risk of VaD. Moreover, we did not find a significant association between FA, FI, or TL and the incidence of VaD (*p* > 0.05) (Figure S4, Table S6).

### 3.4 Causal Effects of the Gut Microbiota on Aging Indicators

**FIGURE 3:**
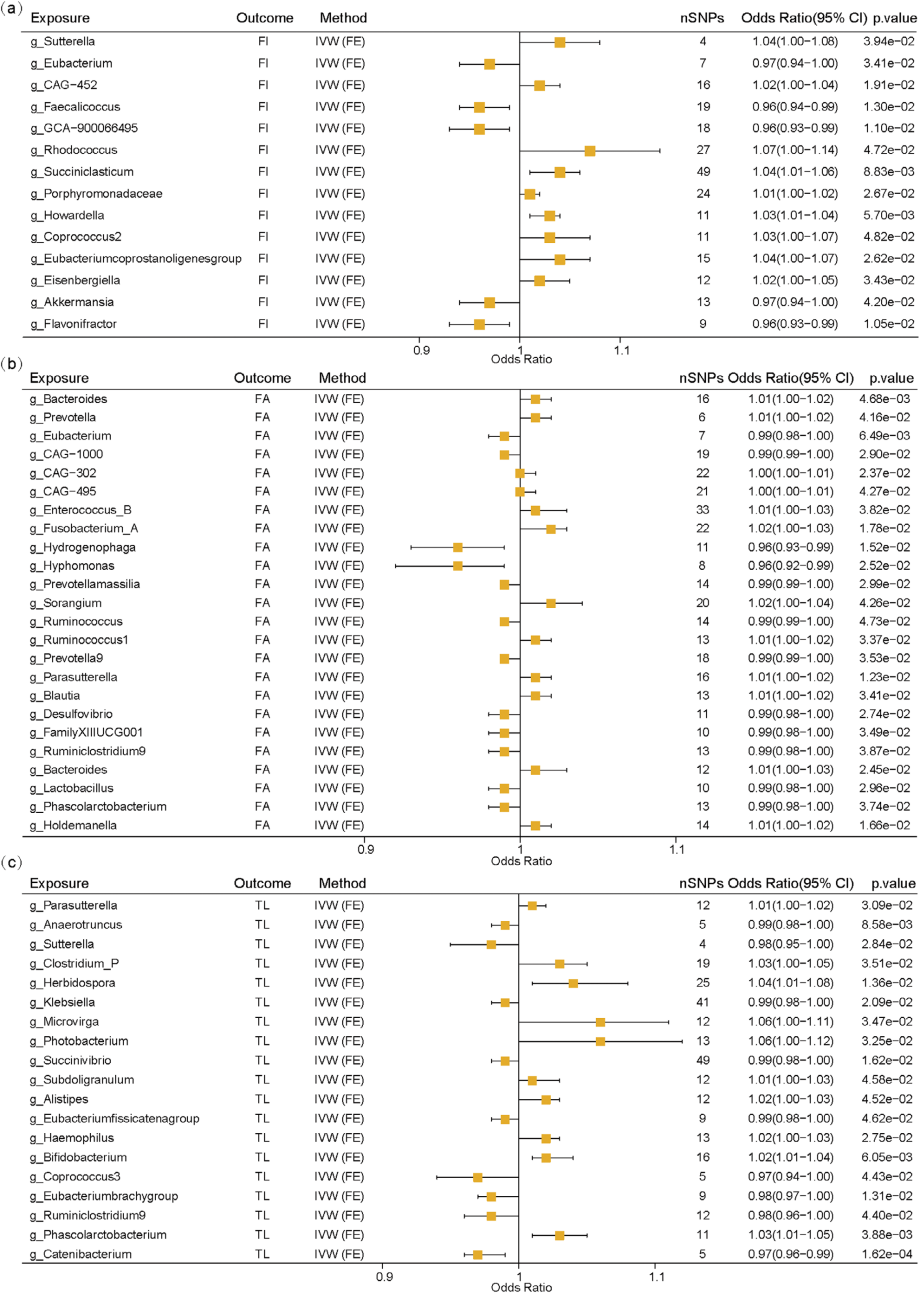
Mendelian randomization results of causal effects of gut mocrobiota on three aging indicators (at genus level). (a) significant results of gut mocrobiota on FI; (b) significant results of gut mocrobiota on FA; (c) significant results of gut mocrobiota on TL. FA, facial aging; FE, fixed effects; FI, frailty index; TL, telomere length.

As shown in Figure 3 and Table S7, gut microbiota were associated with aging indicators. At the genus level, 58, 68, and 58 gut microbiota were associated with FI, FA, and TL, respectively.

### 3.5 Bi‐Directional Causal Effects of Age‐Related Diseases on Gut Microbiota and Aging Indicators

**FIGURE 4:**
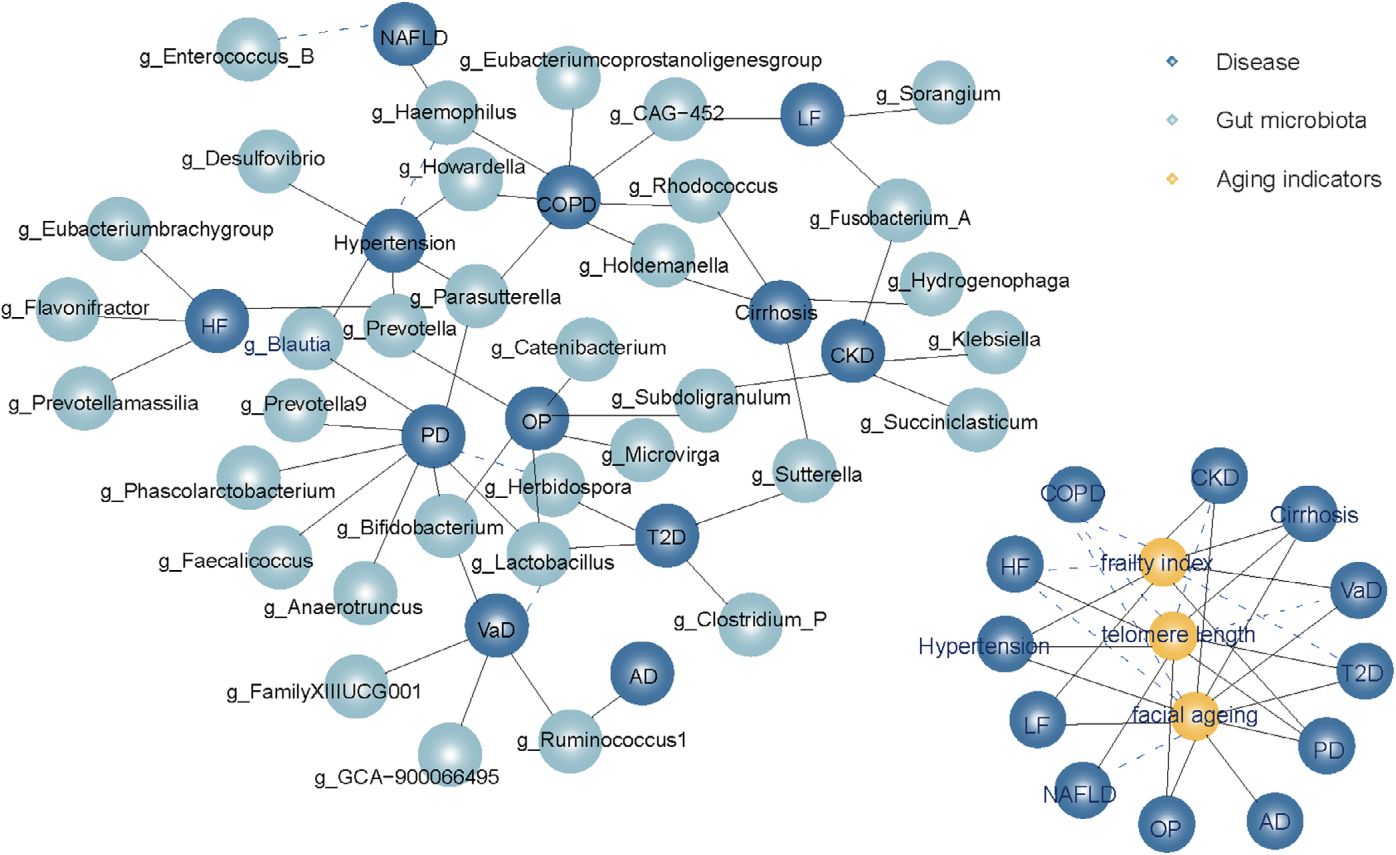
The causal relationships of gut microbiota (at genus level) and aging indicators on age‐related diseases by Mendelian randomization analysis. Black solid line: One‐way causal relationship, gut microbiota/aging indicators on age‐related diseases; black dashed line: Bidirectional causal relationships. AD, Alzheimer's disease; CHD, coronary atherosclerotic heart disease; CKD, chronic kidney disease; COPD, chronic obstructive pulmonary disease; HF, heart failure; LF, liver fibrosis; NAFLD, nonalcoholic fatty liver disease; OP, osteoporosis; PD, Parkinson's disease; T2D, type 2 diabetes; VaD, vascular dementia.

As shown in Table S8, stroke had a causal effect on f_*Clostridiaceae 1* (OR = 0.927, 95% CI = 0.869~0.989, *p* = 0.021) and s_*Lawsonibacter* sp000492175 (OR = 1.027, 95% CI = 1.000~1.053, *p* = 0.042), indicating a bi‐directional causal effect between Stroke and the f_*Clostridiaceae 1/* s_*Lawsonibacter sp000492175*. HF had causal effects on FI (OR = 1.081, 95% CI = 1.033~1.131, *p* = 0.00075), indicating a bi‐directional causal effect between g_*Flavonifractor* and FI. CDK had causal effects on f_*Peptococcaceae* (OR = 1.075, 95% CI = 1.016~1.137, *p* = 0.0117), s_*Bacteroides*_*plebeius* (OR = 1.163, 95% CI = 1.015~1.332, *p* = 0.030), s_*Butyrivibrio*_*crossotus* (OR = 0.841, 95% CI = 0.723~0.979, *p* = 0.025), f_*Lachnospiraceae* (OR = 1.081, 95% CI = 1.014~1.151, *p* = 0.016), o_*Lactobacillales* (OR = 1.105, 95% CI = 1.010~1.208, *p* = 0.292) and TL (OR = 0.986, 95% CI = 0.974 ~ 0.997, *p* = 0.0154), and CHD had a causal effect on s_*Blautia*_*A sp000285855* (OR = 1.027, 95% CI = 1.004 ~ 1.051, *p* = 0.021) and s_*Eisenbergiella sp900066775* (OR = 1.050, 95% CI = 1.012~1.091, *p* = 0.011). Furthermore, VaD had causal effects on g_*Coprobacillus*, and NAFLD had causal effects on five gut microbiota. Additionally, AD, Cirrhosis, COPD, Hypertension, NAFLD, OP, and PD, as shown in Table S8. However, there were no reverse effects among other gut microbiota, aging indicators, and age‐related diseases. Based on these results, we constructed a summary network (Figure 4) to illustrate the causal relationships between multiple gut microbiota (at the genus level), aging indicators, and diseases of aging. The network at the other level is shown in Figure S5.

### 3.6 Sensitivity Analyses

In this study, MR‐Egger tests and Cochran's *Q* tests were used to evaluate the validity of IVs and MR. In the MR‐Egger regression intercept approach and MR‐PRESSO analysis, no significant horizontal pleiotropy (*p*‐value > 0.05) was detected in the outcomes (Table S9). Additionally, the results of the Cochran's *Q* test showed significant heterogeneity (*p* < 0.05) in three outcomes (Table S9). For these outcomes, a random‐effect IVW analysis was performed.

### 3.7 Mediation Analysis of Gut Microbiota, Aging Indicators, and Age‐Related Diseases

In this study, gut microbiota and aging indicators were both causal factors for age‐related diseases. More specifically, it appeared that aging indicators played a mediating role in the pathway between gut microbiota and age‐related diseases. A necessary condition for mediation analysis is the presence of a significant causal relationship between gut microbiota and the mediating factors (aging indicators). These results revealed that there were eight significantly causal effects between gut microbiotas associated with age‐related diseases and aging indicators. Therefore, we used two‐step MR to investigate the mediation effects of FA, FI, and TL. The causal association between f_*Sutterellaceae*/o_*Burkholderiales*/g_*Flavonifractor* and HF was significantly mediated by FI, with a mediation effect of −0.0126 (95% CI: [−0.0255, −0.0031], *p* = 0.0287), −0.0107 (95% CI: [−0.0225, −0.0020], *p* = 0.0435), and − 0.0154 (95% CI: [−0.0326, −0.0028], *p* = 0.0457), respectively. The causal association between f_Erysipelotrichaceae/f_Erysipelatoclostridiaceae/c_Erysipelotrichia/o_*Erysipelotrichales* and Cirrhosis was significantly mediated by TL, with a mediation effect of −0.0126 (95% CI: [−0.0256, −0.0020], *p* = 0.0381), −0.0126 (95% CI: [−0.0256, −0.0020], *p* = 0.0381), −0.0126 (95% CI: [−0.0256, −0.0020], *p* = 0.0381) and − 0.0131 (95% CI: [−0.0150, −0.0028], *p* = 0.0488), respectively. Furthermore, the causal association between s_ *Bifidobacterium breve* and CKD was significantly mediated by TL, with a mediation effect of −0.0035 (95% CI: [−0.0068, −0.0010], *p* = 0.0186) (Table S10).

### 3.8 Gut Bacterial Taxa Closely Related to Human Aging

**FIGURE 5:**
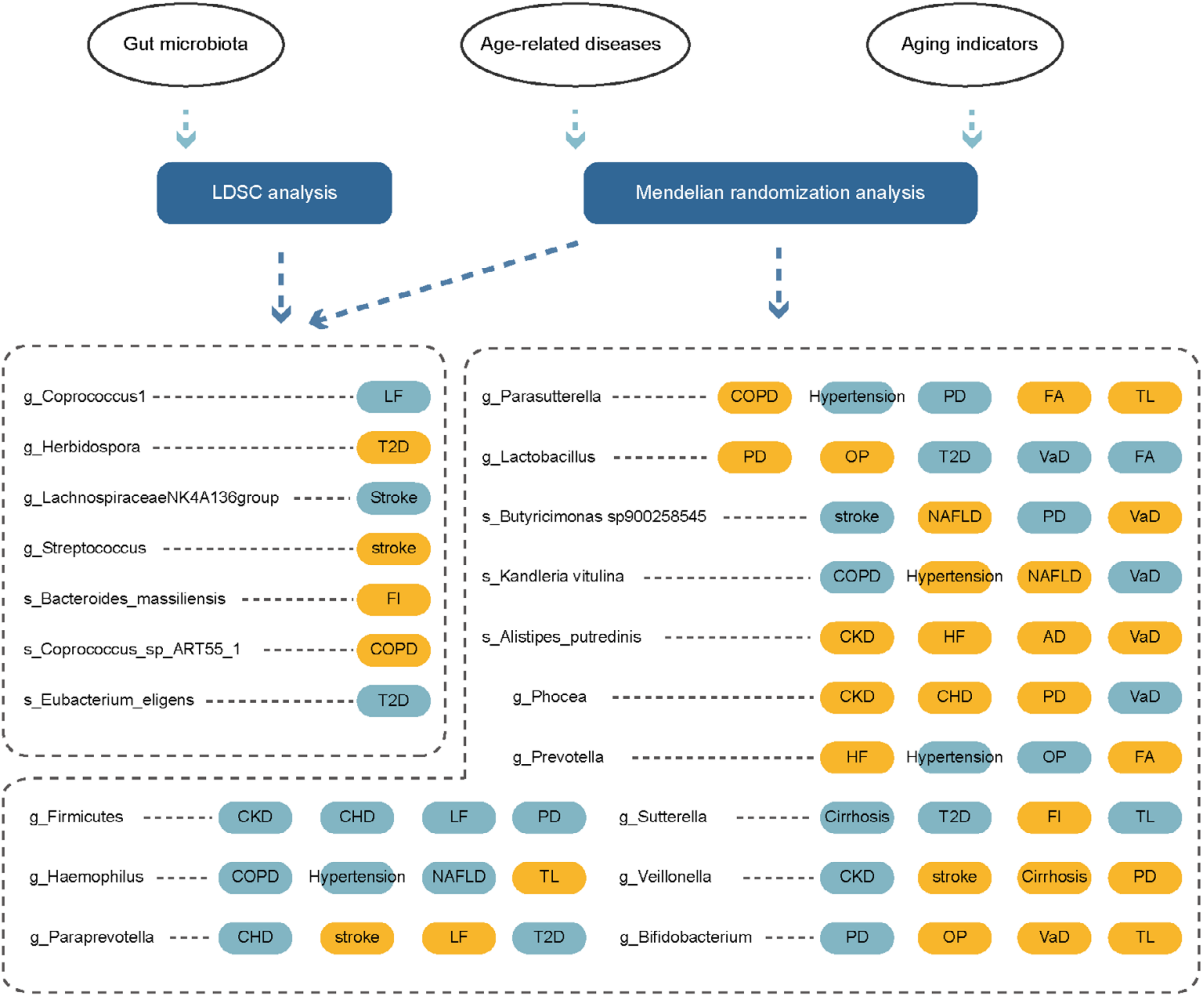
Gut bacterial taxa closely related to human aging. The results of MR were shown in this plot; blue indicates an association between the gut microbe and a reduced risk of the disease/indicator; orange indicates an association between the gut microbe and an increased risk of the indicator/disease. AD, Alzheimer's disease; CHD, coronary atherosclerotic heart disease; CKD, chronic kidney disease; COPD, chronic obstructive pulmonary disease; FA, facial aging; FI, frailty index; HF, heart failure; LF, liver fibrosis; NAFLD, nonalcoholic fatty liver disease; OP, osteoporosis; PD, Parkinson's disease; T2D, type 2 diabetes; TL, telomere length; VaD, vascular dementia.

The results of LDSC and MR analyses indicated that 24 gut microbiota have causal effects on aging indicators or age‐related diseases, such as s_*Bacteroides_massiliensis*, g_*Faecalicoccus*, g_*Faecalibacterium*, g_*Streptococcus*, and g_*Lachnospiraceae NK4A136 group* (Table S11). In this study, gut bacteria with four or more causal relationships with diseases or indicators were considered important bacterial taxa. We identified 22 gut bacterial taxa that are closely associated with human aging. Among them, 14 are significantly causally related to five or more diseases or indicators, as shown in Figure 5 and Table S11.

### 3.9 Performance of Disease Prediction Models Combining MR and LDSC With Machine Learning

**FIGURE 6:**
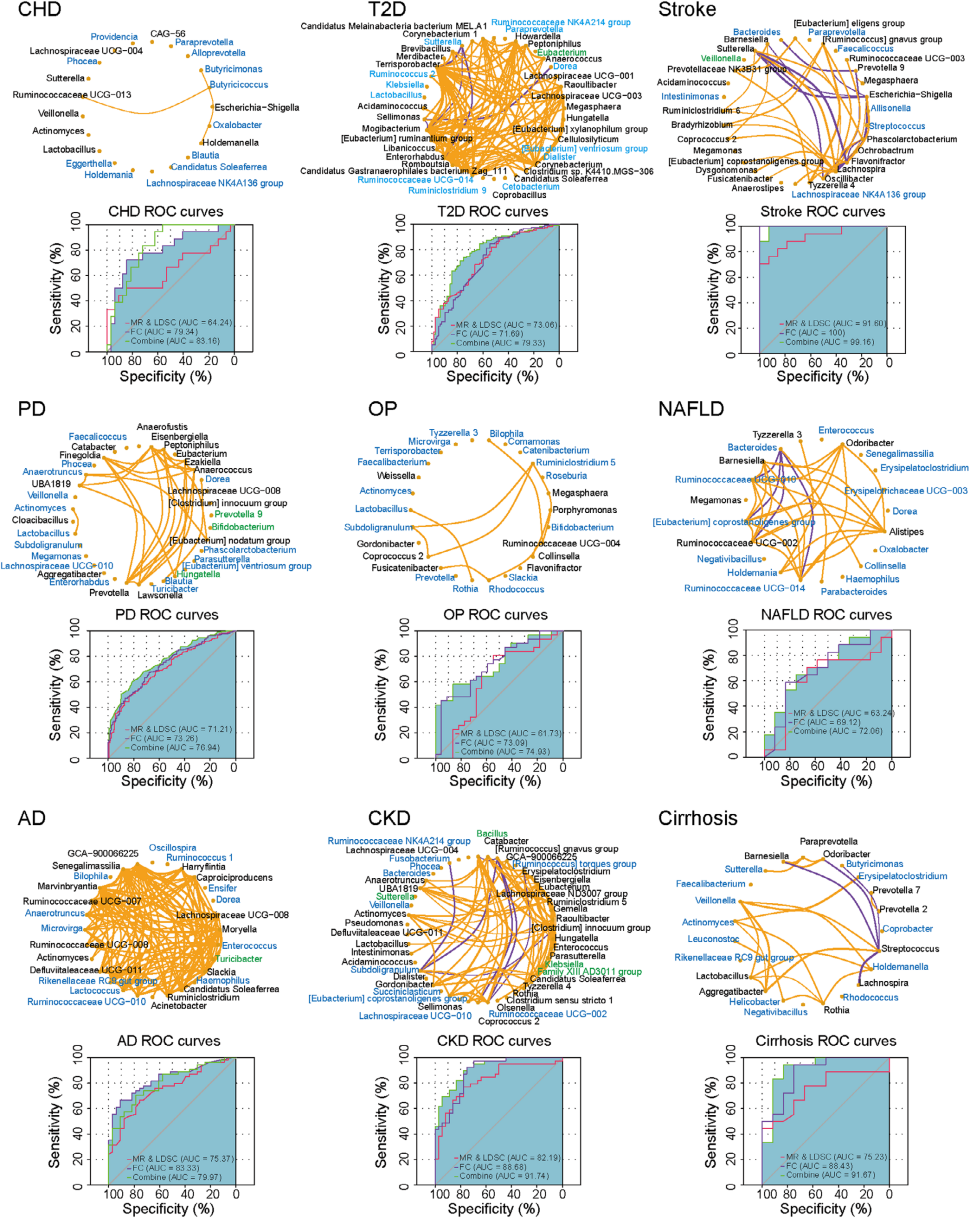
Performance of disease prediction models combining MR and LDSC with machine learning. The network graph shows the gut bacteria obtained by MR and LDSC methods (blue) and those obtained by FC method (black), and Green represents the gut bacteria shared by both methods; Orange lines represent positive correlations, and purple lines represent negative correlations; The thickness of the lines represents the strength of the correlation. AD, Alzheimer's disease; CHD, coronary atherosclerotic heart disease; CKD, chronic kidney disease; COPD, chronic obstructive pulmonary disease; HF, heart failure; LF, liver fibrosis; NAFLD, nonalcoholic fatty liver disease; OP, osteoporosis; PD, Parkinson's disease; T2D, type 2 diabetes; VaD, vascular dementia.

Among the nine constructed disease prediction models, those built using gut microbes identified by MR and LDSC exhibited AUC values ranging from 0.617 to 0.916 (Figure 6), with two diseases achieving AUCs above 0.8. Models constructed using microbes selected based on the FC method showed AUC values between 0.691 and 1.0, with four diseases exceeding an AUC of 0.8. Combining both selection methods generally improved the AUC of disease prediction models, resulting in a range of 0.72 to 0.992. With the exception of the Stroke and AD models, which showed no improvement, all other models demonstrated enhanced performance. Furthermore, correlation network diagrams were constructed for gut microbes identified by both methods, illustrating the correlation patterns among them (Figure 6). Notably, OP and CHD exhibited relatively limited inter‐microbial correlations compared to other diseases.

## 4 Discussion

Given that numerous diseases are closely linked to aging, studies have found connections between the gut microbiota and the onset or progression of these diseases (Wang et al. 2024; Neyrinck et al. 2017). Most research has focused on the relationship between gut microbiota and aging phenotypes, including PhenoAge acceleration, BioAge acceleration, epigenetic age acceleration, DNA methylation, and telomere length (Ye et al. 2024; Xu, Li, et al. 2024; Guolin et al. 2025; Huang et al. 2023). These analyses have consistently demonstrated the intricate causal relationship between gut microbiota and aging. For instance, Guolin et al. (2025) employed bidirectional Mendelian randomization to analyze 207 microbial taxa and seven aging phenotypes, identifying 44 causal relationships between the gut microbiota and aging. Furthermore, animal experiments provided additional validation for the role of *Akkermansia muciniphila* in mitigating aging. However, relatively few studies have incorporated aging‐related diseases to explore the impact of gut microbiota on human aging from a broader perspective. Consequently, we have incorporated aging indicators and a range of age‐related diseases to explore the relationship between the gut microbiota and aging. Based on our current knowledge, this is the first MR study to investigate the causal association between gut microbiota, three aging indicators, and 14 age‐related diseases and assess the mediating effects of indicators. By analyzing the combined genetic data, we found genetic liability to some gut microbiota causally associated with aging. Additionally, we have identified certain gut microbiota that may serve as potential risk factors for age‐related diseases, and aging indicators appear to mediate the causal relationship between these microbiota and age‐related conditions. These results may have implications for public health interventions aimed at facilitating healthy aging.

### 4.1 Genetic Correlation and Causality Analyses Revealed Complex Relationships Between Gut Microbiota and Age‐Related Diseases

LDSC and MR analyses showed that f_*Family XIII* and g_*Lachnospiraceae NK4A136 group* are protective factors for HF and stroke, respectively, while g_*Streptococcus* is a risk factor for stroke. Previous MR studies of HF have not found a causal relationship between f_*Family XIII* and HF (Luo et al. 2022; Pang et al. 2024). This discrepancy is largely due to differences in the datasets used and the screening conditions applied during the analysis. Additionally, the potential causal relationships between g_*Lachnospiraceae NK4A136 group/*g_*Streptococcus* and stroke have been reported (Qin et al. 2023), which are consistent with our findings. Therefore, this study has confirmed the significant causal relationship between gut microbiota and age‐related diseases, as previously indicated by research, and it also contributes to the discovery of potential new causal relationships. Further investigation is required to validate these new findings.

The pathogenesis of different diseases typically exhibits variability, and the functional diversity of gut bacterial taxa leads to inconsistencies in their roles in the onset and progression of various diseases. For instance, in an MR study of multiple cancers, various gut microbial taxa have been found to act as both protective and risk factors (Long et al. 2023). They found that the genus *Tyzzerella 3* is a risk factor for lung adenocarcinoma but a protective factor against colorectal cancer (Long et al. 2023), which illustrates that different species may have divergent effects on the tumor microenvironment (Sivan et al. 2015). In this study, g_*Paraprevotella* is a protective factor for CHD and T2D but a risk factor against stroke and LF; g_ *Lactobacillus* is a protective factor for T2D and VaD but a risk factor against stroke and PD. This suggests that the impact of the same gut microorganisms may vary depending on the disease. Additionally, certain bacterial taxa exhibit similar effects across different diseases and aging indices. As an example, s_*Alistipes_putredinis* is positively associated with the risk of CKD, HF, AD, and VaD; g_*Firmicutes* is negatively associated with the risk of CKD, CHD, LF, and PD. Moreover, among the causal relationships between gut microbiota and age‐related diseases/aging indicators, only five taxa have significant causal associations with four or more diseases/indices. This reflects the limited overlap among gut microbial taxa that potentially exhibit causal relationships with different diseases (Long et al. 2023). In summary, these findings highlight the complexity of the relationship between gut microbiota and the development of various age‐related diseases.

### 4.2 Aging Indices Function as Mediators in the Association Between Gut Microbiota and Age‐Related Diseases

Biological age proxy indicators are commonly used to assess accelerated aging (Chen et al. 2023), facilitating the identification of aging conditions and the timely implementation of appropriate responses. Chen et al. (2023) explicitly elucidated the relationship between overweight and age proxy indicators (FA, FI and TL), emphasizing the potential significance of weight control and overweight treatment in combating accelerated aging. Through the establishment of different exposures and outcomes, this study not only uncovered the potential causal relationships between these age proxy indicators and gut microbiota, as well as diseases, but also explicitly elucidated their mediating role between gut microbiota and diseases. Specifically, TL and FI were found to mediate the causal relationships between gut microbiota and Cirrhosis and HF, respectively.

Telomeres are considered hallmarks of biological aging and play a protective role in the processes of cell death and aging. Typically, extremely short telomeres result in metabolically active cells that are unable to repair damage or divide, leading to aging (Güneşliol et al. 2023). MR analysis confirms the potential causal relationships between TL and o_*Erysipelotrichales*/f_*Erysipelotrichaceae/f_Erysipelatoclostridiaceae*/c_*Erysipelotrichia*, as well as between TL and Cirrhosis. Genetically determined longer TL is associated with a reduced risk of Cirrhosis (Zhu et al. 2023), which is consistent with our findings. Furthermore, in the present study, TL was found to mediate the causal relationship between c_*Erysipelotrichia*/o_*Erysipelotrichales*/f_*Erysipelotrichaceae/f_Erysipelatoclostridiaceae* and Cirrhosis. In addition, TL was found to mediate the causal relationship between s_ *Bifidobacterium breve* and CKD. As a crucial component of the beneficial gut microbiota, Bifidobacteria can participate in the regulation of bile acid synthesis signaling pathways and produce metabolites such as bile acids (Sivamaruthi et al. 2020). Probiotic *Bifidobacterium breve* has been found to develop a normal intestinal microbiota and be involved in protective mechanisms against obesity (Bozzi Cionci et al. 2018). This demonstrates the multifaceted and multimechanistic benefits of Bifidobacteria for human health. Our study further provides additional evidence for the potential benefits of Bifidobacteria in mitigating coronary heart disease, with TL mediating this effect.

Additionally, we have also identified the mediating effect of the FI in the relationship between gut microbiota and disease, primarily within the causal pathway of g_*Flavonifractor/o_Burkholderiales/f_Sutterellaceae* and HF. the pathogenesis of frailty involves pathophysiological processes across multiple systems, and its incidence typically increases with advancing age (Bo et al. 2024). This study found a potential causal relationship between frailty and gut microbiota, as well as HF, which is consistent with findings from previous studies (Xu, Jia, et al. 2024; Bo et al. 2024). In summary, this study not only further corroborates the association between age proxy indicators and gut microbiota as well as age‐related diseases, but also provides evidence that these surrogate indicators are involved in the interplay between gut microbiota and age‐related diseases.

### 4.3 Gut Micro‐Ecologically Regulation May Be an Important Strategy to Promote Healthy Aging

Gut microbiota dysbiosis is thought to contribute to systemic inflammation, which is implicated in the age‐related decline of tissue and immune function (Dejong et al. 2020). Numerous studies have revealed a potential association between gut microbiota and aging. For instance, aging and dysbiosis of the gut microbiota tend to increase systemic changes that promote neuroinflammation (Golomb et al. 2020). Gut bacteria induce senescence of ileal germinal center B cells, leading to a reduction in IgA production and diversity, which in turn contributes to an imbalance in the gut microbiota (Kawamoto and Hara 2024). An MR study investigating the association between gut microbiota and accelerated aging found that increased abundance of *Streptococcus* is associated with accelerated aging (Ye et al. 2024). Notably, our study reveals a causal relationship between the gut microbiome and aging, as evidenced by aging proxy indicators and age‐related diseases. These findings support the notion that gut microbiota dysbiosis is a Hallmarks of aging (Carlos et al. 2023), suggesting that the gut microbiome has the potential to modulate the aging process (Mi Young and Young‐Do 2023). Given the significance of this relationship, microbiota‐based interventions may represent a viable approach for modulating the aging process.

The approaches to delay aging by reshaping the gut microbiota primarily encompass fecal microbiota transplantation (FMT) and probiotics interventions (Zhang et al. 2023). Moderate consumption of probiotics has been shown to harmonize and optimize the gut microbiome, conferring beneficial effects within the body. Numerous experimental studies have corroborated the advantageous role of probiotics in decelerating the aging process (Lin et al. 2021; Wang et al. 2020; Cheng et al. 2022). Heat‐killed *Lactobacillus paracasei* D3‐5 has been shown to reduce age‐related intestinal permeability and inflammation, preventing metabolic dysfunction (Wang et al. 2020). *Lactobacillus plantarum* GKM3 demonstrates advantages in delaying senescence and extending lifespan (Lin et al. 2021). Comparison to probiotic interventions, FMT enables the transfer of an entire microbial community, representing the most direct method to modulating the host's gut microbiota. FMT using microbiota from mice of different age groups demonstrated superior efficacy in promoting mucin production and epithelial barrier integrity, as well as reducing LPS levels, thereby decreasing the likelihood of systemic inflammation induction (Parker et al. 2022). Aged mice receiving gut microbiota transplants from young donors exhibited increased muscle thickness and restored physical fitness, leading to an improved aging phenotype (Kim et al. 2022). In addition, experiments in progeroid mice have shown the efficacy of fecal microbiota transplantation in improving both healthspan and lifespan (Bárcena et al. 2019). Therefore, gut microbiota interventions represent a promising therapeutic avenue for anti‐aging. This study has elucidated the potential relationship between specific gut microbial taxa and aging from both correlative and causal perspectives. These findings may serve as a basis for microbiota‐targeted interventions and contribute to a holistic approach for promoting anti‐aging strategies.

### 4.4 Genetic Correlation and Causal Relationship Analyses Can Serve as Preliminary Screening Methods for Disease Prediction Models

Gut microbes are increasingly being used as non‐invasive biomarkers for disease pre‐screening and have demonstrated promising independent diagnostic performance across various diseases (Li, Liu, et al. 2023). Machine learning, as a powerful tool, has found numerous applications in the detection of health and disease microbial signatures as well as in the prediction of health status (Zeller et al. 2014; Tap et al. 2017; Salim et al. 2023; Aryal et al. 2020). This study integrated LDSC and MR results with machine learning to construct prediction models for nine age‐related diseases, achieving an average AUC of 0.731. Although this performance is lower than that of models based on conventional biomarker selection (average AUC = 0.808), this discrepancy is likely attributable to the fact that the GWAS data primarily originated from European populations, whereas the data used for model construction predominantly comprised gut microbiota data from Asian populations. Diet is considered a major factor influencing gut microbiota composition (Sonia and María Ángeles 2021), and dietary patterns typically vary across geographical regions, suggesting potential differences in gut microbiota profiles between cohorts from different regions. Li, Liu, et al. (2023) validated gut microbial diagnostic models for 20 diseases and observed that within‐cohort prediction performance (average AUC = 0.77) was superior to cross‐cohort prediction performance (average AUC = 0.64). This further highlights the influence of cohort‐specific factors on the utility of gut microbes as independent predictors of disease. Nevertheless, the models constructed in this study based on LDSC and MR analyses exhibited good predictive performance across different cohorts, demonstrating the potential of leveraging genetic correlations and causality for identifying gut microbial markers that can be applied in disease prediction.

Integrating the results of LDSC and MR with fold change (FC)‐based selection generally enhanced the predictive performance of the models (average AUC = 0.832). Although the improvement was not observed in the stroke and AD models, the final AUC and FC results showed no significant differences. Overall, incorporating genetic correlation and causality as supplementary criteria for biomarker selection appears to mitigate, to some extent, the impact of cross‐regional and cross‐cohort factors on model applicability and effectively improve prediction performance. Therefore, in clinical applications such as disease prediction and prognostic assessment, the use of causal inference algorithms to identify potential biomarkers remains of significant value, as it can enhance the predictive accuracy of models. Such models may serve as clinical decision support tools, helping to reduce diagnostic errors and improve efficiency. However, the limitations of available datasets continue to pose major challenges in the identification of robust biomarkers. With the continuous accumulation of clinical data, the advantages of current analytical methods and models are expected to become increasingly evident.

### 4.5 Research Limitations

It should be noted that various factors, such as diet and environmental influences, have been shown to affect the gut microbiota. Studies have reported the impact of different dietary patterns on the gut microbiome, and dietary interventions have been explored as a strategy to modulate microbial communities (Sonia and María Ángeles 2021; Solch et al. 2022). Additionally, environmental factors, including pollution, occupational environment, and residential location, can also influence individual health and microbial composition (Yuanxiang et al. 2017). These factors represent potential confounders in our analysis. Pavlović et al. (2024) have noted that, for predictive purposes, confounding might not significantly compromise model performance and may even enhance predictive accuracy. However, when the objective is to elucidate underlying biological mechanisms or estimate causal effects, confounders should be carefully controlled. In this study, due to the complexity of analyzing multiple diseases and the extensive nature of potential confounders, we did not explicitly adjust for these factors within the causal inference framework. Nevertheless, this approach is unlikely to compromise the predictive performance of our models (Pavlović et al. 2024). However, it should be emphasized that confounding factors cannot be disregarded. In future studies focusing on specific causal relationships, further analyses will be required to ensure the accuracy and robustness of causal inferences.

Of course, we must acknowledge the limitations of this study. First, given that the majority of the GWAS summary data utilized in this study are derived from European populations, this may introduce bias estimates and affect the universality of the findings. Second, the thresholds during the screening process affect the proportion of SNPs utilized. This implies that some genetic instrumental variables may be overlooked, leading to the omission of some results. Finally, the study focused on a limited number of 14 diseases, lacking the comprehensive scope to fully explore the complexities of age‐related diseases. Future research should address these limitations.

## 5 Conclusion

In this study, bidirectional MR and LDSC algorithms were used to screen gut microbiota as potential biomarkers for age‐related diseases, and to evaluate the causal relationship between gut microbiota, three aging indicators, and a range of age‐related diseases. This analysis elucidated the mediating role of certain aging indicators. Machine learning models were also built to evaluate the effectiveness of biomarkers in the prediction of age‐related diseases. These results may provide new insights into the mechanisms of gut microbiota/aging indicators‐mediated age‐related disease development. Overall, this study demonstrates the potential of genetic correlation and causal inference analyses as tools for identifying age‐related disease biomarkers. This approach can facilitate early prediction of age‐related diseases and provide supporting information for the development of targeted interventions to promote healthy aging.

## Author Contributions

The authors' responsibilities were as follows: Chunrong Lu: study design, data analysis, manuscript drafting, and manuscript revision; Xiaojun Wang: study design, data analysis, manuscript drafting; Xiaochun Chen: study design, data analysis, contribution to discussion and manuscript revision, and supervision; Pengpeng Ye, Tao Qin, Jianqun Liu, Shuai Wang, and Weifei Luo: data analysis. All authors read and approved the final manuscript.

## Conflicts of Interest

The authors declare no conflicts of interest.

## Supporting information


Appendix S1

## Data Availability

Data that support the findings of this study but are not included in the article or Supporting Information files are available from the corresponding author upon reasonable request.
