# Supplementary material for: Causal Analysis Between Gut Microbes, Aging Indicator, and Age‐Related Disease, Involving the Discovery and Validation of Biomarkers
Source: Aging Cell. 2025 Apr 9;24(7):e70057. doi: 10.1111/acel.70057 (PMC12266770; doi:10.1111/acel.70057)
Supplement: Supplementary file 1 — Appendix S1 [file ACEL-24-e70057-s001.zip › acel70057-sup-0001-Supplementarymaterial1.pdf]

Supplementary material 1

Causal analysis between gut microbes, aging indicator and age-related disease, involving the discovery and validation of biomarkers

Chunrong Lu[1#], Xiaojun Wang[1,2#], Xiaochun Chen[1,2#*], Tao Qin[1], Pengpeng Ye[1,2], Jianqun Liu[1], Shuai Wang[1,3], and Weifei Luo[1,2]

To assess the discriminative ability of gut microbiota in disease diagnostics, we constructed supervised machine learning (ML) models using the PyCaret package. Features for these models consisted of bacterial taxa abundances, specifically those demonstrating significant associations with diseases in prior Mendelian Randomization (MR) and Linkage Disequilibrium Score Regression (LDSC) analyses.

Bacterial sequence data, obtained from NCBI (https://www.ncbi.nlm.nih.gov/), underwent processing with QIIME 2 to derive abundance data[1]. Taxonomic classification was achieved using a naive Bayes classifier against the SILVA 132 reference database. To mitigate the influence of low-abundance taxa, bacteria present in fewer than 10% of samples were excluded from further analysis. The sample composition for the nine diseases included in this study is detailed in **Table e1**.

Before building the model, we combined the corresponding datasets for nine diseases, if multiple datasets were available. Because microbiome sequencing data is mostly zero-bloated, we used the MMUPHin workflow[2], which is specifically designed for microbiome OTU profiling, to reduce variance caused by batch effects. In this workflow, permutational multivariate analysis of variance (PERMANOVA) was used to quantify variance, and principal coordinates analyses (PCoA) was used to visualize batch effect correction results. The distance metric for PCoA was based on Bray-Curtis dissimilarity (**Figure e1**). PERMANOVA and PCoA were implemented using the adonis2 and vegdist function from the vegan package, respectively[3]. The stroke disease contained only one dataset and thus did not require batch effect correction. After correcting for batch effects, we combined all datasets for each disease and

used the merged data to construct ML models.

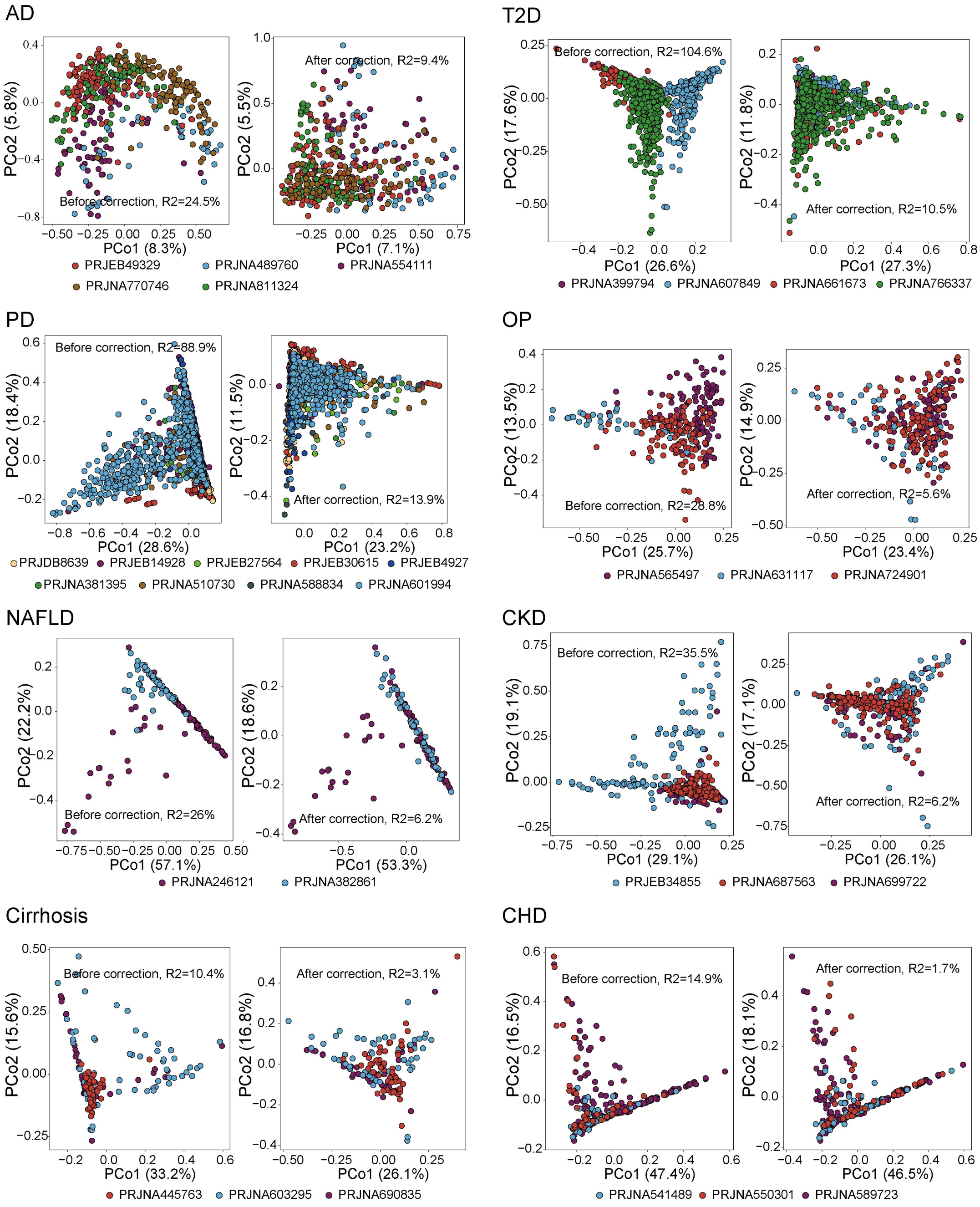

Figure e1 The PCoA of the eight diseases was based on genus-level Bray-Curtis dissimilarity. For each disease, two plots were generated: one before and one after batch correction, with studies represented by different colors. R2 values were calculated using permutational multivariate analysis of variance (PERMANOVA, n = 999 permutations) and reflect the proportion of variance attributable to study and batch effects. The R2 value reduced from 24.5% to 9.4% in AD, 14.9% to 1.7% in CHD, 10.4% to 3.1% in Cirrhosis, 35.5% to 6.2% in CKD, 26% to 6.2% in NAFLD, 28.8% to

5.6% in OP, 88.9% to 13.9% in PD, 104.6% to 10.5% in T2D, respectively.

Generally, differentially abundant bacteria are used to construct ML models. We employ fold change (FC) and *p*-value to measure differential bacteria between disease and healthy groups, where *p*-values are derived from the Wilcoxon rank-sum test. When a bacterium's logFC and *p*-value meet specified thresholds (logFC > 1 and *p*-value < 0.05 for CKD, Stroke, and T2D; logFC > 0.5 and *p*-value < 0.05 for NAFLD, Cirrhosis, and CHD, different thresholds were used due to the limited number of microbiota in some diseases), we define this bacterium as a differentially abundant bacterium (DAB), and all DABs are used to build ML models (FC method)[4]. Additionally, we use gut bacteria taxa identified from LDSC and MR analyses to construct ML models (MR&LDSC method). Finally, we combine gut bacteria from both methods to construct ML models for different diseases and assess the effectiveness of different methods for disease classification (Combine method). The gut bacteria used for the different models are shown in **Table e2**.

During the model-building process, all samples were randomly divided into training and validation sets at a ratio of 8:2. The training set was used to build predictive models, and the validation set was used to evaluate their performance. Using the PyCaret package (with a fixed random seed to ensure model reproducibility), a total of 60 ML models were developed based on the training data. The performance of each model was preliminarily assessed using five cross-validation. The model with the highest accuracy was selected as the optimal model for subsequent validation[5, 6]. Finally, the diagnostic performance was evaluated using the AUC value obtained on the validation set. To optimize model performance, hyperparameters were fine-tuned over 200 iterations using a random search strategy. The receiver operating characteristic (ROC) curve was plotted based on the optimal model's performance on the validation set, and an area under the curve (AUC) threshold greater than 0.7 was considered to indicate diagnostic capability[7]. The entire process is illustrated in **Figure e2**.

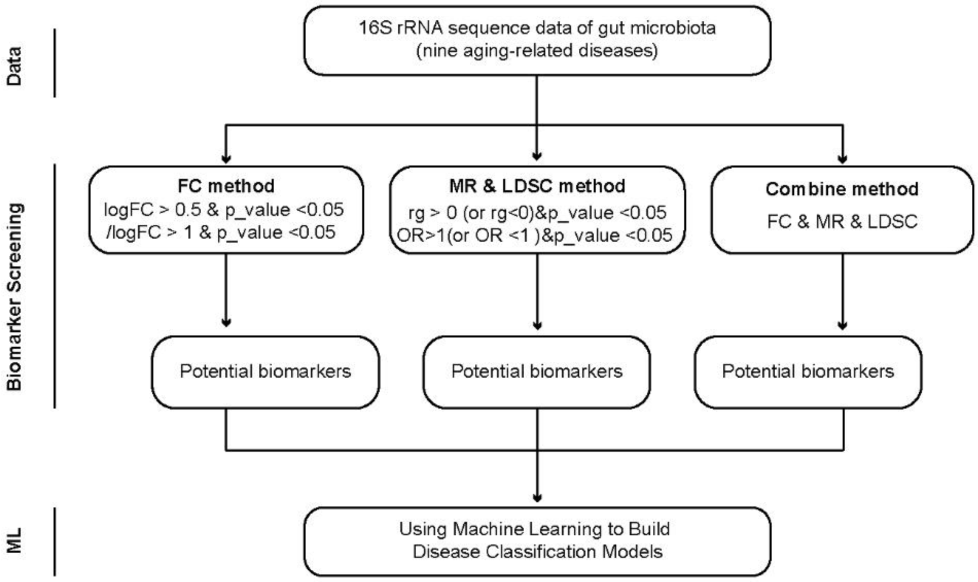

Figure e2 Disease classification model construction process. MR, Mendelian Randomization; LDSC, Linkage Disequilibrium Score Regression; ML, machine learning

Table e1 Source of the 16S rRNA SEQUANCEdata

Disease	Access number	Description	Region
CHD	PRJNA589723	Include 109 CHD patients and 49 controls	China
	PRJNA550301	Include 18CHD patients and 23 controls	China
	PRJNA541489	Include 24CHD patients and 23 controls	China
CKD	PRJNA699722	Include 108 CKD patients	China
	PRJEB34855	Include 71 CKD patients and 46 controls	South Korea
	PRJNA687563	Include 16 CKD patients and 134 controls	China
NAFLD	PRJNA246121	Include 53 NAFLD patients and 32 controls	China
	PRJNA382861	Include 31 NAFLD patients and 25 controls	China
T2D	PRJNA399794	Include 11 T2D patients and 19 controls	Brazilian
	PRJNA607849	Include 98 T2D patients and 19 controls	Nigeria
	PRJNA661673	Include 60 T2D patients and 60 controls	China
	PRJNA766337	Include 383 T2D patients and 114 controls	Japan
Cirrhosis	PRJNA690835	Include 16 Cirrhosis patients and 22 controls	China
	PRJNA603295	Include 36 Cirrhosis patients and 19 controls	China
	PRJNA445763	Include 36 Cirrhosis patients and 20 controls	China
Stroke	PRJNA734855	Include 83 Stroke patients and 70 controls	China
AD	PRJNA554111	Include 37 AD patients and 36 controls	China
	PRJNA489760	Include 30 AD patients and 30 controls	China
	PRJEB49329	Include 116 AD patients	Holland
	PRJNA811324	Include 43 AD patients and 41 controls	Kazakhstan
	PRJNA770746	Include 54 AD patients and 45 controls	Canada
PD	PRJEB14928	Include 28 PD patients and 30 controls	Germany
	PRJEB27564	Include 136 PD patients and 130 controls	Finland
	PRJEB30615	Include 124 PD patients and 92 controls	Germany
	PRJNA381395	Include 26 PD patients and 38 controls	Europe
	PRJNA510730	Include 118 PD patients and 84 controls	Italy
	PRJNA588834	Include 69 PD patients and 64 controls	China

	PRJEB4927	Include 74 PD patients and 74 controls	Finland
	PRJDB8639	Include 223 PD patients and 137 controls	Japan
	PRJNA601994	Include 323 PD patients and 184 controls	America
OP	PRJNA565497	Include 85 OP patients and 28 controls	China
	PRJNA724901	Include 65 OP patients and 43 controls	China
	PRJNA631117	Include 23 OP patients and 17 controls	China

Note: T2D, diabetes mellitus type 2; CKD, chronic kidney disease; CVD cardiovascular disease; CHD, coronary heart disease; HF, heart failure; COPD, chronic obstructive pulmonary disease; LD, liver disease; LF, liver fibrosis; NAFLD, nonalcoholic fatty liver disease; AD, Alzheimer's disease; PD, Parkinson's disease; OP, Osteoporosis; FA, facial aging; FI, frailty index; TL, telomere length; MR, Mendelian Randomization; LDSC, Linkage Disequilibrium Score Regression

Table e2 Gut microbiota used for modelling by different methods

Disease	Methods	Gut bacteria
CHD	FC	Escherichia-Shigella, CAG-56, Lachnospiraceae UCG-004, Sutterella, Ruminococcaceae UCG-013, Veillonella, Actinomyces, Lactobacillus, Holdemanella
	MR & LDSC	Paraprevotella, Providencia, Phocea, Butyricicoccus, Lachnospiraceae NK4A136 group, Candidatus Soleaferrea, Eggerthella, Oxalobacter, Holdemania, Alloprevotella, Blautia, Butyricimonas
	Combine	Escherichia-Shigella, CAG-56, Lachnospiraceae UCG-004, Sutterella, Ruminococcaceae UCG-013, Veillonella, Actinomyces, Lactobacillus, Holdemanella, Butyricicoccus, Butyricimonas, Alloprevotella, Paraprevotella, Providencia, Phocea, Eggerthella, Holdemania, Lachnospiraceae NK4A136 group, Candidatus Soleaferrea, Blautia, Oxalobacter
CKD	FC	[Clostridium] innocuum group, Raoultibacter, Gemella, Ruminiclostridium 5, Lachnospiraceae ND3007 group, Eubacterium, Eisenbergiella, Erysipelatoclostridium, GCA-900066225, [Ruminococcus] gnavus group, Catabacter, Lachnospiraceae UCG-004, Anaerotruncus, UBA1819, Actinomyces, Pseudomonas, Defluviitaleaceae UCG-011, Lactobacillus, Intestinimonas, Acidaminococcus, Dialister, Gordonibacter, Sellimonas, Coprococcus 2, Olsenella, Clostridium sensu stricto 1, Rothia, Tyzzerella 4, Candidatus Soleaferrea, Parasutterella, Enterococcus, Hungatella, Family XIII AD3011 group, Klebsiella
	MR & LDSC	Klebsiella, Ruminococcaceae UCG-002, Subdoligranulum, Sutterella, Ruminococcaceae NK4A214 group, Lachnospiraceae UCG-010, [Eubacterium] coprostanoligenes group, Phocea, Succiniclasticum, Bacillus, Fusobacterium, [Ruminococcus] torques group, Veillonella, Family XIII AD3011 group, Bacteroides

	Combine	[Clostridium] innocuum group, Raoultibacter, Gemella, Ruminiclostridium 5, Lachnospiraceae ND3007 group, Eubacterium, Eisenbergiella, Erysipelatoclostridium, GCA-900066225, [Ruminococcus] gnavus group, Catabacter, Lachnospiraceae UCG-004, Anaerotruncus, UBA1819, Actinomyces, Pseudomonas, Defluviitaleaceae UCG-011, Lactobacillus, Intestinimonas, Acidaminococcus, Dialister, Gordonibacter, Sellimonas, Coprococcus 2, Olsenella, Clostridium sensu stricto 1, Rothia, Tyzzerella 4, Candidatus Soleaferrea, Parasutterella, Enterococcus, Hungatella, [Ruminococcus] torques group, Ruminococcaceae NK4A214 group, Bacillus, Fusobacterium, Phocea, Bacteroides, Sutterella, Veillonella, Subdoligranulum, Succiniclasticum, [Eubacterium] coprostanoligenes group, Lachnospiraceae UCG-010, Ruminococcaceae UCG-002, Family XIII AD3011 group, Klebsiella
NAFLD	FC	Odoribacter, Tyzzerella 3, Barnesiella, Megamonas, Ruminococcaceae UCG-002, Alistipes
	MR & LDSC	[Eubacterium] coprostanoligenes group, Senegalimassilia, Collinsella, Haemophilus, Erysipelotrichaceae UCG-003, Parabacteroides, Oxalobacter, Holdemania, Negativibacillus, Erysipelatoclostridium, Dorea, Ruminococcaceae UCG-014, Bacteroides, Enterococcus, Ruminococcaceae UCG-010
	Combine	Odoribacter, Tyzzerella 3, Barnesiella, Megamonas, Ruminococcaceae UCG-002, Alistipes, Dorea, Erysipelotrichaceae UCG-003, Erysipelatoclostridium, Senegalimassilia, Enterococcus, Bacteroides, Ruminococcaceae UCG-010, [Eubacterium] coprostanoligenes group, Negativibacillus, Holdemania, Ruminococcaceae UCG-014, Parabacteroides, Haemophilus, Collinsella, Oxalobacter

T2D	FC	Megasphaera, Lachnospiraceae UCG-003, Raoultibacter, Lachnospiraceae UCG-001, Anaerococcus, Peptoniphilus, Howardella, Candidatus Melainabacteria bacterium MEL.A1, Corynebacterium 1, Brevibacillus, Merdibacter, Terrisporobacter, Acidaminococcus, Sellimonas, Mogibacterium, [Eubacterium] ruminantium group, Libanicoccus, Enterorhabdus, Romboutsia, Candidatus Gastranaerophilales bacterium Zag_111, Coprobacillus, Candidatus Soleaferrea, Clostridium sp. K4410.MGS-306, Corynebacterium, Cellulosilyticum, [Eubacterium] xylanophilum group, Hungatella
	MR & LDSC	Cetobacterium, Ruminococcaceae NK4A214 group, Klebsiella, Eubacterium, Dorea, Dialister, Paraprevotella, Ruminiclostridium 9, [Eubacterium] ventriosum group, Ruminococcus 2, Sutterella, Ruminococcaceae UCG-014, Lactobacillus
	Combine	Megasphaera, Lachnospiraceae UCG-003, Raoultibacter, Lachnospiraceae UCG-001, Anaerococcus, Peptoniphilus, Howardella, Candidatus Melainabacteria bacterium MEL.A1, Corynebacterium 1, Brevibacillus, Merdibacter, Terrisporobacter, Acidaminococcus, Sellimonas, Mogibacterium, [Eubacterium] ruminantium group, Libanicoccus, Enterorhabdus, Romboutsia, Candidatus Gastranaerophilales bacterium Zag_111, Coprobacillus, Candidatus Soleaferrea, Clostridium sp. K4410.MGS-306, Corynebacterium, Cellulosilyticum, [Eubacterium] xylanophilum group, Hungatella, Dorea, Eubacterium, Paraprevotella, Ruminococcaceae NK4A214 group, Sutterella, Ruminococcus 2, Klebsiella, Lactobacillus, Ruminococcaceae UCG-014, Ruminiclostridium 9, Cetobacterium, Dialister, [Eubacterium] ventriosum group
Cirrhosis	FC	Prevotella 2, Prevotella 7, Odoribacter, Paraprevotella, Barnesiella, Lactobacillus, Aggregatibacter, Rothia, Lachnospira, Streptococcus
	MR & LDSC	Holdemanella, Erysipelatoclostridium, Sutterella, Negativibacillus, Coprobacter, Rhodococcus, Rikenellaceae RC9 gut group, Helicobacter, Faecalibacterium, Butyricimonas, Leuconostoc, Actinomyces, Veillonella

	Combine	Prevotella 2, Prevotella 7, Odoribacter, Paraprevotella, Barnesiella, Lactobacillus, Aggregatibacter, Rothia, Lachnospira, Streptococcus, Coprobacter, Erysipelatoclostridium, Butyricimonas, Sutterella, Faecalibacterium, Leuconostoc, Rikenellaceae RC9 gut group, Helicobacter, Negativibacillus, Rhodococcus, Holdemanella, Veillonella, Actinomyces
Stroke	FC	Escherichia-Shigella, Megasphaera, Prevotella 9, Ruminococcaceae UCG-003, [Ruminococcus] gnavus group, [Eubacterium] eligens group, Sutterella, Prevotellaceae NK3B31 group, Acidaminococcus, Ruminiclostridium 6, Bradyrhizobium, Coprococcus 2, Megamonas, [Eubacterium] coprostanoligenes group, Dysgonomonas, Fusicatenibacter, Anaerostipes, Tyzzerella 4, Oscillibacter, Lachnospira, Flavonifractor, Ochrobactrum, Phascolarctobacterium, Barnesiella, Veillonella
	MR & LDSC	Allisonella, Faecalicoccus, Intestinimonas, Streptococcus, Paraprevotella, Bacteroides, Lachnospiraceae NK4A136 group, Veillonella
	Combine	Escherichia-Shigella, Megasphaera, Prevotella 9, Ruminococcaceae UCG-003, [Ruminococcus] gnavus group, [Eubacterium] eligens group, Sutterella, Prevotellaceae NK3B31 group, Acidaminococcus, Ruminiclostridium 6, Bradyrhizobium, Coprococcus 2, Megamonas, [Eubacterium] coprostanoligenes group, Dysgonomonas, Fusicatenibacter, Anaerostipes, Tyzzerella 4, Oscillibacter, Lachnospira, Flavonifractor, Ochrobactrum, Phascolarctobacterium, Allisonella, Faecalicoccus, Paraprevotella, Bacteroides, Intestinimonas, Lachnospiraceae NK4A136 group, Streptococcus, Barnesiella, Veillonella
AD	FC	Moryella, Lachnospiraceae UCG-008, Caproiciproducens, Harryflintia, GCA-900066225, Senegalimassilia, Marvinbryantia, Ruminococcaceae UCG-007, Ruminococcaceae UCG-008, Actinomyces, Defluviitaleaceae UCG-011, Acinetobacter, Ruminiclostridium, Candidatus Soleaferrea, Slackia, Turicibacter

	MR & LDSC	Oscillospira, Turicibacter, Haemophilus, Rikenellaceae RC9 gut group, Ruminococcus 1, Lactococcus, Ruminococcaceae UCG-010, Dorea, Enterococcus, Anaerotruncus, Ensifer, Bilophila, Microvirga
	Combine	Moryella, Lachnospiraceae UCG-008, Caproiciproducens, Harryflintia, GCA-900066225, Senegalimassilia, Marvinbryantia, Ruminococcaceae UCG-007, Ruminococcaceae UCG-008, Actinomyces, Defluviitaleaceae UCG-011, Acinetobacter, Ruminiclostridium, Candidatus Soleaferrea, Slackia, Dorea, Ensifer, Ruminococcus 1, Oscillospira, Bilophila, Anaerotruncus, Microvirga, Rikenellaceae RC9 gut group, Lactococcus, Ruminococcaceae UCG-010, Haemophilus, Enterococcus, Turicibacter
PD	FC	[Clostridium] innocuum group, Lachnospiraceae UCG-008, Anaerococcus, Ezakiella, Eubacterium, Peptoniphilus, Eisenbergiella, Anaerofustis, Catabacter, Finegoldia, UBA1819, Cloacibacillus, Aggregatibacter, Prevotella, Lawsonella, [Eubacterium] nodatum group, Prevotella 9, Hungatella, Bifidobacterium
	MR & LDSC	Lactobacillus, Actinomyces, Phascolarctobacterium, Parasutterella, Phocea, Veillonella, Megamonas, Anaerotruncus, Bifidobacterium, Lachnospiraceae UCG-010, Faecalicoccus, [Eubacterium] ventriosum group, Enterorhabdus, Dorea, Blautia, Subdoligranulum, Turicibacter, Hungatella, Prevotella 9
	Combine	[Clostridium] innocuum group, Lachnospiraceae UCG-008, Anaerococcus, Ezakiella, Eubacterium, Peptoniphilus, Eisenbergiella, Anaerofustis, Catabacter, Finegoldia, UBA1819, Cloacibacillus, Aggregatibacter, Prevotella, Lawsonella, [Eubacterium] nodatum group, Dorea, Faecalicoccus, Phocea, Anaerotruncus, Veillonella, Actinomyces, Lactobacillus, Subdoligranulum, Megamonas, Lachnospiraceae UCG-010, Enterorhabdus, Turicibacter, Blautia, [Eubacterium] ventriosum group, Parasutterella, Phascolarctobacterium, Prevotella 9, Hungatella, Bifidobacterium

OP	FC	Porphyromonas, Megasphaera, Weissella, Coprococcus 2, Fusicatenibacter, Rothia, Rhodococcus, Flavonifractor, Collinsella, Ruminococcaceae UCG-004
	MR & LDSC	Actinomyces, Terrisporobacter, Ruminiclostridium 5, Gordonibacter, Slackia, Tyzzerella 3, Faecalibacterium, Subdoligranulum, Catenibacterium, Bilophila, Microvirga, Comamonas, Roseburia, Bifidobacterium, Prevotella, Lactobacillus
	Combine	Porphyromonas, Megasphaera, Weissella, Coprococcus 2, Fusicatenibacter, Rothia, Rhodococcus, Flavonifractor, Collinsella, Ruminococcaceae UCG-004, Roseburia, Ruminiclostridium 5, Catenibacterium, Comamonas, Bilophila, Tyzzerella 3, Microvirga, Terrisporobacter, Faecalibacterium, Actinomyces, Lactobacillus, Subdoligranulum, Gordonibacter, Prevotella, Slackia, Bifidobacterium

Note: T2D, diabetes mellitus type 2; CKD, chronic kidney disease; CVD cardiovascular disease; CHD, coronary heart disease; HF, heart failure; COPD, chronic obstructive pulmonary disease; AD, Alzheimer's disease; PD, Parkinson's disease; OP, Osteoporosis; FA, facial aging; FI, frailty index; TL, telomere length

References

[1] Usyk M, Zolnik C P, Castle P E, et al. Cervicovaginal microbiome and natural history of HPV in a longitudinal study[J]. PLoS pathogens, 2020, 16(3): e1008376.

[2] Mei Z, Wang F, Bhosle A, et al. Strain-specific gut microbial signatures in type 2 diabetes identified in a cross-cohort analysis of 8,117 metagenomes[J]. Nat Med, 2024, 30(8): 2265-2276.

[3] Nishiwaki H, Ueyama J, Ito M, et al. Meta-analysis of shotgun sequencing of gut microbiota in Parkinson's disease[J]. NPJ Parkinsons Dis, 2024, 10(1): 106.

[4] Erawijantari P P, Mizutani S, Shiroma H, et al. Influence of gastrectomy for gastric cancer treatment on faecal microbiome and metabolome profiles[J]. Gut, 2020, 69(8): 1404-1415.

[5] Lee S, Portlock T, Le Chatelier E, et al. Global compositional and functional states of the human gut microbiome in health and disease[J]. Genome Res, 2024, 34(6): 967-978.

[6] Bao Z, Yang Z, Sun R, et al. Predicting host health status through an integrated machine learning framework: insights from healthy gut microbiome aging trajectory[J]. Scientific reports, 2024, 14(1): 31143.

[7] Chen H C, Liu Y W, Chang K C, et al. Gut butyrate-producers confer post-infarction cardiac protection[J]. Nature communications, 2023, 14(1): 7249.
