# Supplementary material for: Causal Analysis Between Gut Microbes, Aging Indicator, and Age‐Related Disease, Involving the Discovery and Validation of Biomarkers
Source: Aging Cell. 2025 Apr 9;24(7):e70057. doi: 10.1111/acel.70057 (PMC12266770; doi:10.1111/acel.70057)
Supplement: Supplementary file 1 — Appendix S1 [file ACEL-24-e70057-s001.zip › acel70057-sup-0002-Supplementarymaterial2.pdf]

Supplementary material 2

Causal analysis between gut microbes, aging indicator and age-related disease, involving the discovery and validation of biomarkers

Chunrong Lu[1#], Xiaojun Wang[1,2#], Xiaochun Chen[1,2#*], Tao Qin[1], Pengpeng Ye[1,2], Jianqun Liu[1], Shuai Wang[1,3], and Weifei Luo[1,2]

Figure captions

Figure S1 Circular heat map of suggestive genetic correlations between gut microbes, aging indicators and age-related diseases; Prefix "p_/c_/o_/f_/g_" represents phylum/class/order/family/genus, respectively; * represents P <0.05; rg, genetic correlation

Figure S2 The genetic correlations between aging indicators and aging diseases; Solid and hollow represent P <0.05 and P >0.05, respectively

Figure S3 Causal effect results of gut microbiota on aging indicators and age-related diseases

Figure S4 The causal relationships between gut microbiota (phylum to family level), ageing indicators and ageing-related diseases by Mendelian randomisation analysis; black solid line: gut microbiota on ageing-related diseases; black dashed line: ageing-related diseases on gut microbiota

Figure S5 Gut bacterial taxa closely related to human aging (at species levels)

Table S1 Overview of the source of gut microbita, diseases and aging indicators data

Table S2 The genetic correlations between gut microbes, aging indicators, and age-related diseases

Table S3 The selected SNPs of gut microbiota at $P < 1 \times 10^{-5}$

Table S4 The selected SNPs of FA, FI, and TL at $P < 5 \times 10^{-8}$

Table S5 The causal effects of gut microbiota on age-related diseases

Table S6 The causal effects of aging indicators on multiple age-related diseases

Table S7 The causal effects of gut microbiota on aging indicators

Table S8 Reverse causal effects of age-related diseases on gut microbiota and aging

indicators

Table S9 Results of sensitivity analyses

Table S10 Results of Mediation analysis

Table S11 Gut bacterial taxa closely related to human aging

Figure S1 Circular heat map of suggestive genetic correlations between gut microbes, aging indicators and age-related diseases; Prefix “p_/c_/o_/f_/g_” represents phylum/class/order/family/genus, respectively; *represents P <0.05; rg, genetic correlation; CKD, Chronic kidney disease; COPD, Chronic obstructive pulmonary disease; CHD, Coronary heart disease; HF, Heart failure; T2D, type 2 Diabetes; NAFLD, Nonalcoholic fatty liver disease; LF, Liver fibrosis; AD, Alzheimer's disease; PD, Parkinson's disease; OP, Osteoporosis; VaD, Vascular dementia; FA, facial aging; FI, frailty index; TL, telomere length

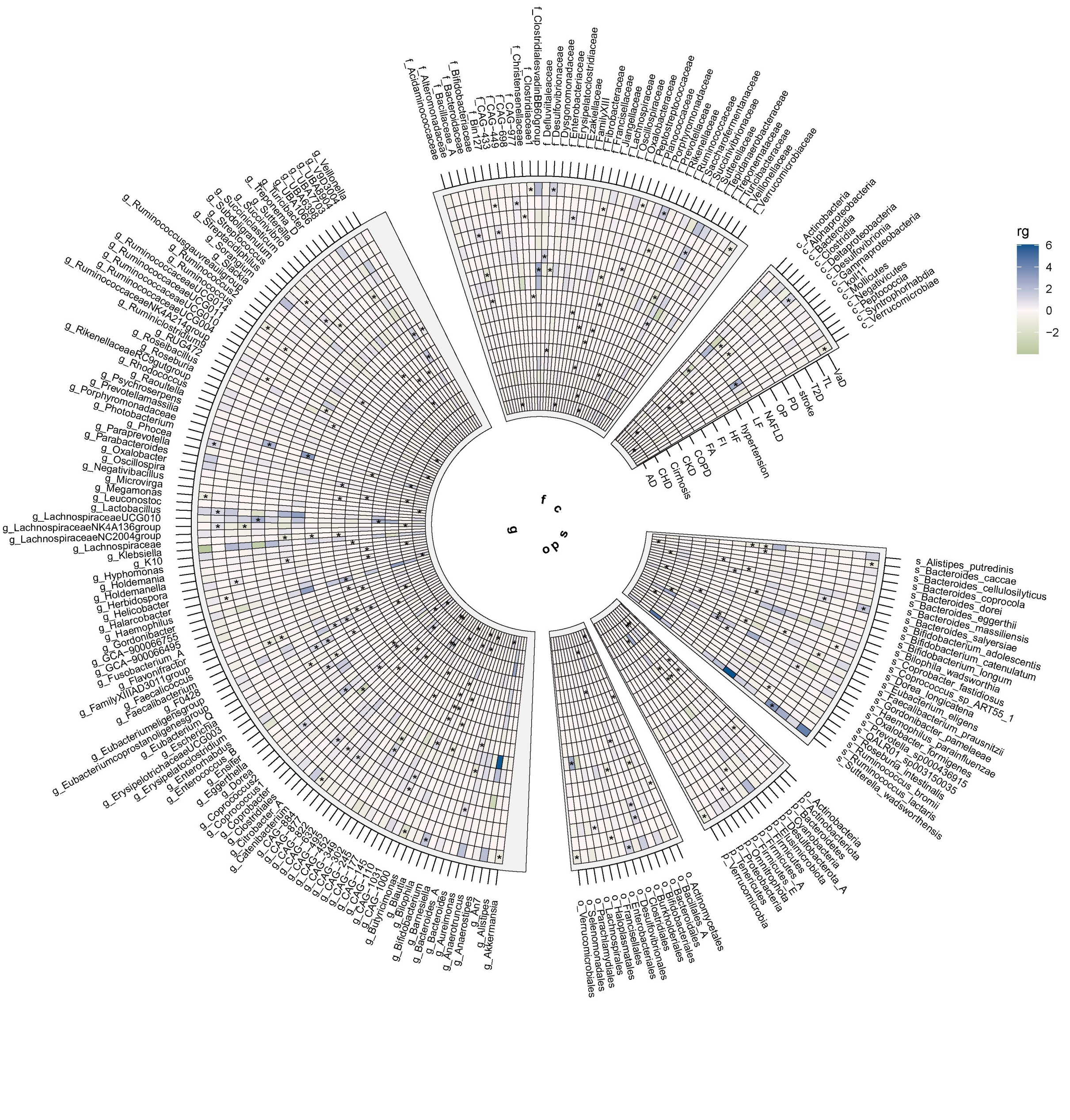

Figure S2 The genetic correlations between aging indicators and aging diseases; Solid and hollow represent *P* <0.05 and *P* >0.05 respectively; CKD, Chronic kidney disease; COPD, Chronic obstructive pulmonary disease; CHD, Coronary heart disease; HF, Heart failure; T2D, type 2 Diabetes; NAFLD, Nonalcoholic fatty liver disease; LF, Liver fibrosis; AD, Alzheimer's disease; PD, Parkinson's disease; OP, Osteoporosis; VaD, Vascular dementia; FA, facial aging; FI, frailty index; TL, telomere length

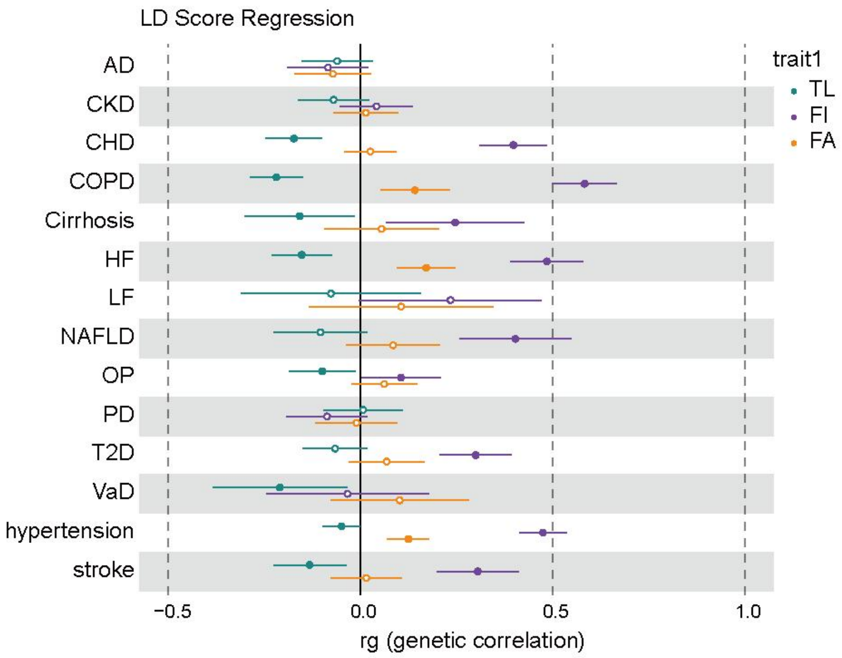

Figure S3 Causal effect results of gut microbiota on age-related diseases. Associations with a *P*-value < 0.05 are represented by dots. The size of the dots corresponds the effect size. Blue dots signify a positive causal effect on the outcome, while green dots indicate a negative causal effect; CKD, Chronic kidney disease; COPD, Chronic obstructive pulmonary disease; CHD, Coronary heart disease; HF, Heart failure; T2D, type 2 Diabetes; NAFLD, Nonalcoholic fatty liver disease; LF, Liver fibrosis; AD, Alzheimer's disease; PD, Parkinson's disease; OP, Osteoporosis; VaD, Vascular dementia

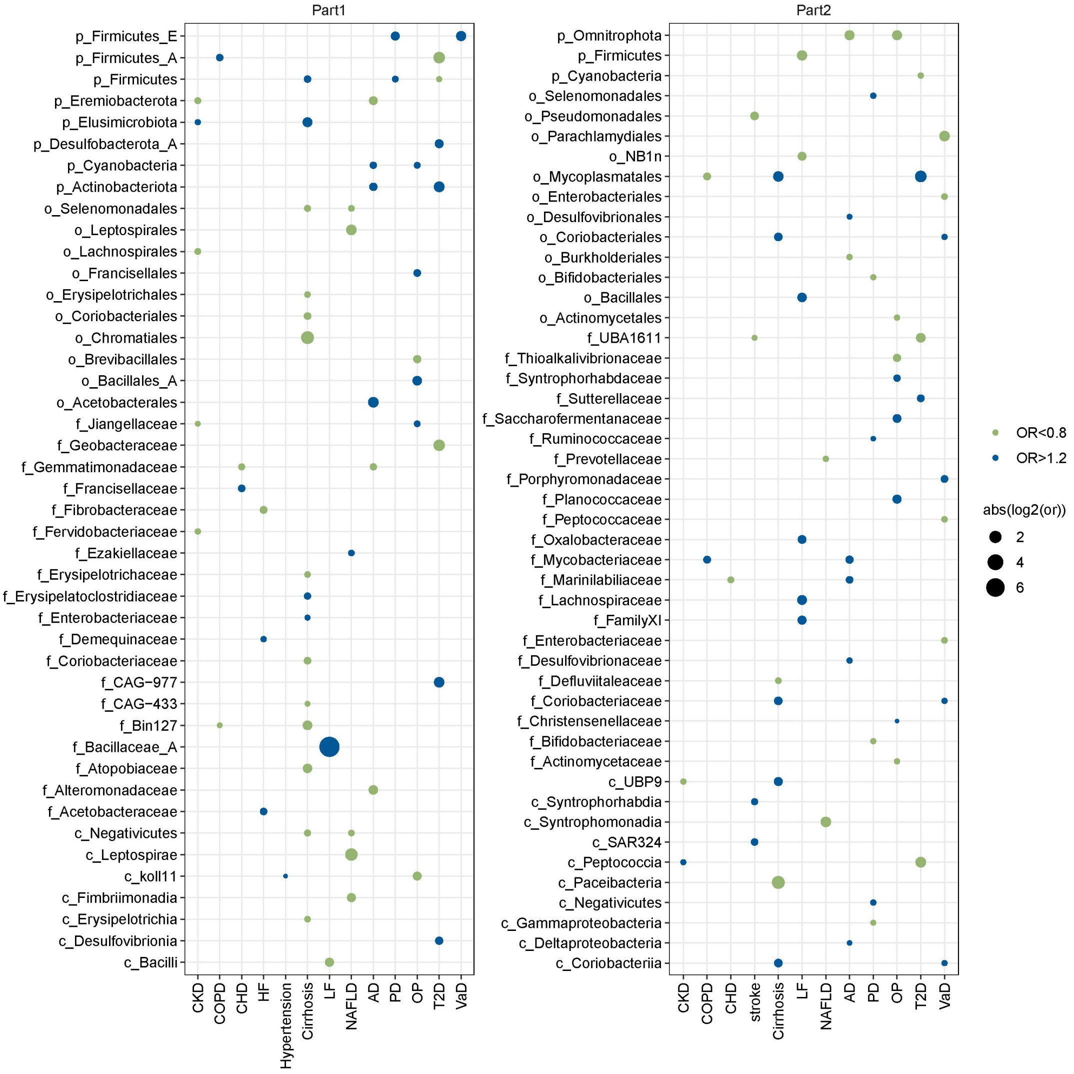

Figure S4 Significant causal effects of aging indicators on age-related diseases. IVW, inverse-variance weighted; FE, fixed effects; MRE, multiplicative random effects; CKD, Chronic kidney disease; COPD, Chronic obstructive pulmonary disease; CHD, Coronary heart disease; HF, Heart failure; T2D, type 2 Diabetes; NAFLD, Nonalcoholic fatty liver disease; LF, Liver fibrosis; AD, Alzheimer's disease; PD, Parkinson's disease; OP, Osteoporosis; VaD, Vascular dementia

exposure	outcome	method	nSNPs	Odds Ratio(95% CI)	p.value
facial ageing	CKD	IVW (FE)	79	0.69(0.53−0.89)	3.99e−03
facial ageing	Cirrhosis	IVW (FE)	82	0.51(0.19−1.36)	1.79e−01
facial ageing	LF	IVW (FE)	82	0.22(0.01−3.41)	2.78e−01
facial ageing	NAFLD	IVW (FE)	82	0.54(0.27−1.07)	7.97e−02
facial ageing	PD	IVW (FE)	82	0.67(0.40−1.15)	1.48e−01
facial ageing	OP	IVW (FE)	82	1.24(0.83−1.86)	2.97e−01
facial ageing	VaD	IVW (FE)	81	0.95(0.48−1.91)	8.95e−01
frailty index	CKD	IVW (FE)	13	0.93(0.72−1.19)	5.50e−01
frailty index	CHD	IVW (FE)	13	1.62(1.20−2.19)	1.69e−03
frailty index	HF	IVW (FE)	14	1.42(1.16−1.74)	6.01e−04
frailty index	stroke	IVW (FE)	16	1.29(0.99−1.68)	6.19e−02
frailty index	Cirrhosis	IVW (FE)	14	0.87(0.36−2.09)	7.59e−01
frailty index	NAFLD	IVW (FE)	14	1.14(0.62−2.10)	6.70e−01
frailty index	AD	IVW (FE)	14	0.72(0.49−1.04)	7.62e−02
frailty index	T2D	IVW (FE)	6	1.93(0.89−4.19)	9.72e−02
frailty index	VaD	IVW (FE)	14	1.81(0.97−3.36)	6.03e−02
telomere length	CKD	IVW (FE)	191	0.88(0.82−0.94)	1.64e−04
telomere length	stroke	IVW (FE)	182	1.03(0.96−1.11)	4.36e−01
telomere length	Cirrhosis	IVW (FE)	198	0.57(0.45−0.72)	4.96e−06
telomere length	LF	IVW (FE)	199	0.74(0.37−1.46)	3.85e−01
telomere length	NAFLD	IVW (FE)	199	0.96(0.81−1.14)	6.35e−01
telomere length	PD	IVW (FE)	199	1.15(1.01−1.32)	3.28e−02
telomere length	T2D	IVW (MRE)	46	0.88(0.66−1.18)	4.09e−01

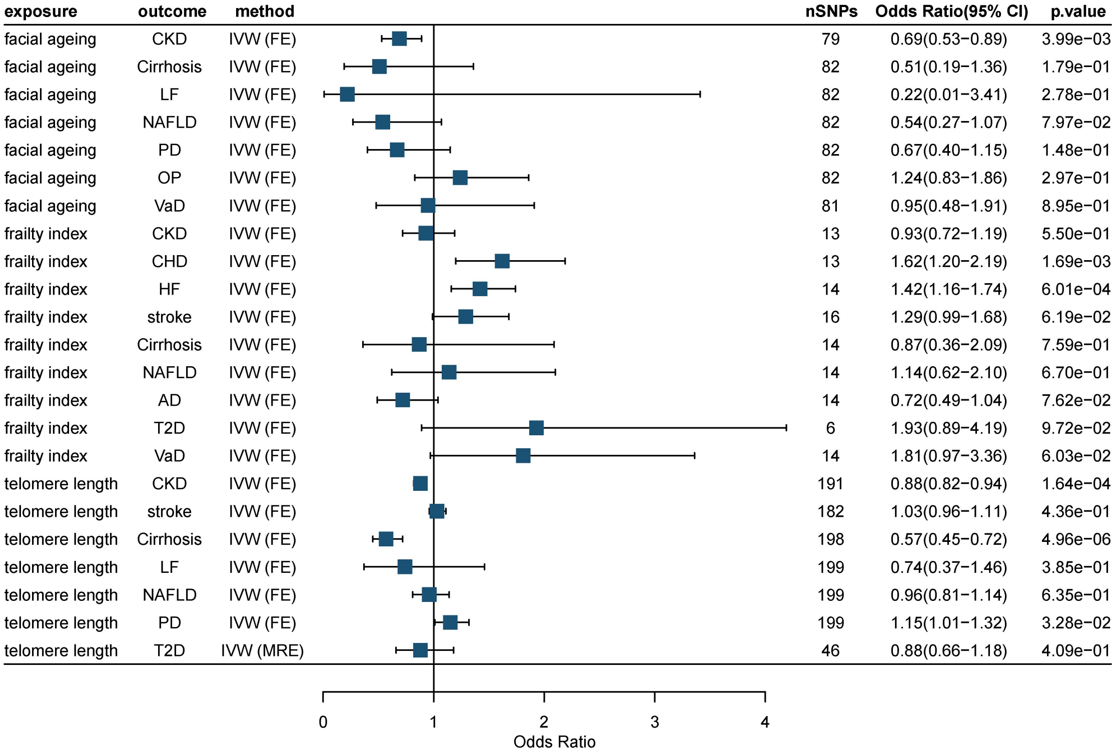

Figure S5 The causal relationships of gut microbiota (at species level) and ageing indicators on ageing-related diseases by Mendelian randomisation analysis; black solid line: one-way causal relationship, gut microbiota/ageing indicators on ageing-related diseases; black dashed line: bidirectional causal relationships; CKD, Chronic kidney disease; COPD, Chronic obstructive pulmonary disease; CHD, Coronary heart disease; HF, Heart failure; T2D, type 2 Diabetes; NAFLD, Nonalcoholic fatty liver disease; LF, Liver fibrosis; AD, Alzheimer's disease; PD, Parkinson's disease; OP, Osteoporosis; VaD, Vascular dementia

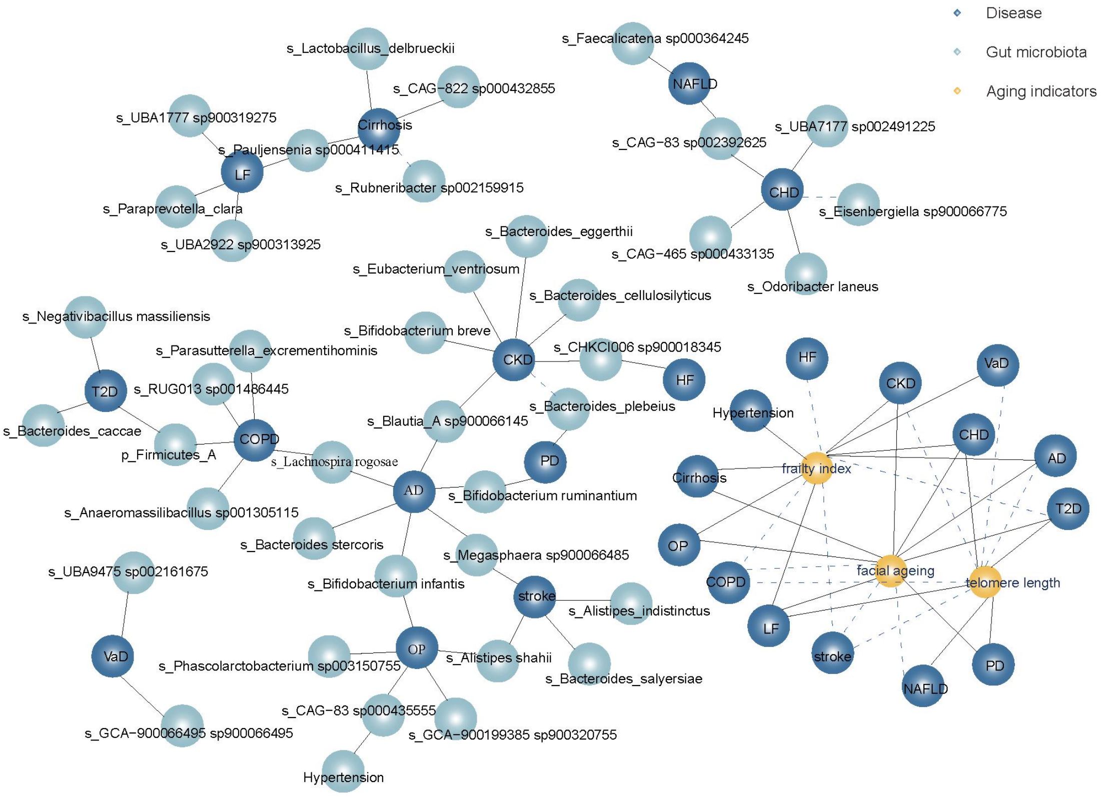

Table S1 Overview of the source of gut microbiota, age-related diseases and aging indicators data

Phenotypes	Source	Year of publication	Codes*, references or ICD codes	Sample size	Cases (n)	Controls (n)	Age scope
**Gut microbiota**							
Gut microbiota	https://mibiogen.gcc.rug.nl	2021	MiBioGen consortium	18,340	NA	NA	NA
	https://www.ebi.ac.uk/gwas/publications/33462482	2022	FINRISK 2002	5,959	NA	NA	NA
	https://www.ebi.ac.uk/gwas/publications/35115689	2021	German individuals	8,956	NA	NA	NA
	https://www.ebi.ac.uk/gwas/publications/35115690	2022	DMP	7,738	NA	NA	NA
**age-related Disease**							
CKD	GWAS Catalogs	2019	GCST008065	625,219	64,164	561,055	54(median)
COPD	GWAS Catalog	2018	GCST90018807	635,145	17,547	617,598	63(mean)
CHD	Open GWAS	2015	CARDIoGRAMplusC4D	184,305	60,801	123,504	50+ (mean)
HF	GWAS Catalog	2020	GCST009541	977,323	47,309	930,014	60+ (mean)

stroke	EBI database	2018	ebi-GCST006908	440,328	34,217	406,111	60+ (mean)
Hypertension	https://r12.finngen.fi/	2024	I9_HYPTENS	500,264	154,630	345,634	59 (median)
T2D	https://www.diagram-consortium.org	2012	DIAGRAM (BMI unadjusted T2D)	898,130	74,124	824,006	50+ (mean)
NAFLD	https://r12.finngen.fi/	2024	NAFLD	500,348	3,504	496,844	52 (median)
LF	https://r12.finngen.fi/	2024	FIBROLIV	495,017	214	494,803	59 (median)
Cirrhosis	https://r12.finngen.fi/	2024	CHIRHEP_NAS	496,506	1,703	494,803	66 (median)
AD	GWAS Catalog	2019	GCST007511	63,926	21,982	41,944	65+ (late-onset)
PD	https://r12.finngen.fi/	2024	G6_PARKINSON	500,348	5,861	494,487	71 (median)
OP	https://r12.finngen.fi/	2024	M13_OSTEOPOROSIS	483,725	10,461	473,264	76 (median)
VaD	https://r12.finngen.fi/	2024	F5_VASCDEM	479,108	3,624	475,484	82 (median)
**Aging indicator**							
FA	UK Biobank	2018	ukb-b-2148	423,999	NA	NA	NA
FI	GWAS Catalog	2021	GCST90020053	175,226	NA	NA	NA
TL	Open GWAS	2021	ieu-b-4879	472,174	NA	472,174	NA

Note: CKD, Chronic kidney disease; COPD, Chronic obstructive pulmonary disease; CHD, Coronary heart disease; HF, Heart failure; T2D, type 2 Diabetes; NAFLD, Nonalcoholic fatty liver disease; LF, Liver fibrosis; AD, Alzheimer's disease; PD, Parkinson's disease; OP, Osteoporosis; VaD, Vascular dementia; FA, Facial ageing; FI, Frailty index; TL, Telomere length
